# Timeliness of Routine Vaccination, Catch-Up Completion, and Immune Function in Chinese Children with Special Healthcare Needs: A Retrospective Cohort Study

**DOI:** 10.3390/vaccines14020149

**Published:** 2026-01-31

**Authors:** Yuyuan Zeng, Xihan Li, Yu Tian, Yuming Liu, Jianhong Wang, Qi An, Chuanyu Yang, Bo Zhou, Lili Zhang, Yangmu Huang, Lin Wang

**Affiliations:** 1 Capital Institute of Pediatrics, Chinese Academy of Medical Sciences & Peking Union Medical College, Beijing 100020, China; b2024032010@student.pumc.edu.cn (Y.Z.);; 2 Department of Child Health Care, Capital Institute of Pediatrics, Capital Center for Children’s Health, Capital Medical University, Beijing 100020, China; 3 School of Public Health, Capital Medical University, Beijing 100069, China; 4 Department of Global Health, School of Public Health, Peking University, Beijing 100191, China

**Keywords:** children with special healthcare needs (CSHCNs), vaccines, national immunization program (NIP), immunization consultation

## Abstract

Background: Children with special healthcare needs (CSHCNs) face persistent barriers to timely immunization in China, but comparative evidence across disease groups and vaccines, and data on immune function, are limited. Methods: We conducted a retrospective cohort study linking electronic medical records, vaccination records, and a structured telephone and questionnaire follow-up. We estimated timely vaccination by National Immunization Program (NIP) dose definitions, assessed catch-up completion at follow-up, and compared cellular/humoral/complement immune indices with published pediatric reference ranges. Group differences used ANOVA/Kruskal–Wallis and chi-square (χ^2^)/Fisher’s exact tests with Bonferroni correction. Results: Timely vaccination was lower than the national healthy child benchmarks for all NIP vaccines (all *p* < 0.001); the Japanese encephalitis virus (JE; 24.0%) and measles-containing vaccine (MCV; 25.9%) had the lowest timely completion. A subset of CSHCNs did not receive recommended catch-up vaccinations, primarily due to persistent caregivers’ concern and point of vaccination (POV) staff’s hesitancy. Delays clustered in neonatal/perinatal disorders for Bacillus Calmette–Guérin (BCG) and hepatitis B vaccine, dose 1 (HepB1). Catch-up completion was highest for hepatitis B vaccine, dose 3 (HepB3) (86.3%) and BCG (81.8%), and lowest for the diphtheria and tetanus vaccine (DT) (49.4%); MCV2 completion was particularly low in hematological diseases. Immunoglobulin A (IgA) and immunoglobulin G (IgG) concentrations were significantly lower in neonatal/perinatal disorders and infectious disease groups versus neurological and immune disorder groups (*p* < 0.05). No severe adverse events were reported after catch-up. Conclusions: CSHCNs in China face substantial barriers to timely NIP immunization. Timeliness and catch-up vary substantially by vaccine and underlying condition; neonatal/perinatal disorders contribute disproportionately to early-life delays. Disease-specific guidance, strengthened POV–specialist clinic coordination, immunological monitoring, and supportive policies could improve the vaccination coverage and effectiveness in this vulnerable population.

## 1. Introduction

China’s National Immunization Program (NIP, launched in 1978) distinguishes NIP from non-NIP vaccines and schedules multi-dose series across childhood (Table 1) [1]. Since its establishment, the NIP has been a cornerstone of China’s vaccine-preventable disease control and has expanded in scope over time [2]. Recent national reports indicate coverage of >98.7% for NIP doses in healthy children [3,4].

Children with special healthcare needs (CSHCNs) are those who require additional services or support because of chronic conditions, disabilities, or other medical complexities that can affect vaccine safety and efficacy. Although clinical guidelines for the vaccination of CSHCNs exist, CSHCNs often experience delayed or deferred vaccination. In China, heterogeneous service capacity and the lack of sufficiently detailed operational guidance across conditions have been reported as important system-level barriers to consistent immunization delivery for children with medical conditions [5]. International studies show substantially lower vaccination rates than in the general population [6,7,8]. For example, in premature infants aged 0–6 months, timely vaccination rates were only 17.02% for BCG, 18.72% for the hepatitis B vaccine (HepB), 6.38% for the diphtheria, tetanus, and acellular pertussis combined vaccine (DTaP), 13.62% for the poliomyelitis vaccine (PV), and 14.89% for the group A meningococcal polysaccharide vaccine (MPSV-A) [7]. A previous study conducted in China showed that the overall timely vaccination rate was only 5.9% among CSHCNs [9]. Emerging evidence from vaccination consultation clinic settings in China further suggests that specialist recommendations can stratify CSHCNs into “routine continuation”, “conditional/partial vaccination”, or “temporary deferral” pathways, but also underscores the variability in recommendations across conditions and the need for clearer, standardized implementation approaches [10,11]. Given higher risks of vaccine-preventable diseases in under-immunized CSHCNs, identifying drivers of delay and safe catch-up pathways is a policy priority. Recent China-focused policy/implementation discussions likewise emphasize actionable strategies to reduce missed opportunities and improve catch-up for CSHCNs [12]. Catch-up vaccination in CSHCNs is often influenced not only by access or scheduling factors, but also by clinical decision-making and caregiver concerns regarding vaccine safety and expected effectiveness in the context of underlying immune status. Profiling T- and B-cell compartments and complement function provides objective evidence of immune competence and infection susceptibility, which vaccination consultation clinicians use to inform risk assessment and guide catch-up decisions.

Evidence is scarce on how timeliness differs by underlying condition and vaccine, which children complete catch-up after specialist advice, and how immune indices compare across disease groups. We addressed these gaps by linking clinical, immunization, and follow-up data by quantifying vaccine-specific delays and catch-up completion, stratified by disease group and evaluating immune indices across disease groups. To our knowledge, this is the first study in China to simultaneously examine disease-group-specific, vaccine dose-level timeliness patterns alongside immune indices in CSHCNs, with the goal of enhancing vaccination coverage through evidence-based policy and practice for this population.

## 2. Materials and Methods

### 2.1. Study Population

This retrospective cohort study is reported in accordance with the STROBE (Strengthening the Reporting of Observational Studies in Epidemiology) guidelines. We conducted this study in an authoritative tertiary pediatric immunization consultation clinic. Data were collected from consecutive children who presented for vaccination consultation between August 2019 and April 2025 (inclusive). Inclusion criteria: Children presenting for immunization consultation due to factors potentially affecting vaccination (including underlying medical conditions and/or clinician- or caregiver-raised concerns). Exclusion criteria: Children were excluded only when the medical chart did not contain sufficient diagnostic documentation to determine a primary underlying condition category. Follow-up ended on 30 June 2025 (Figure S1).

### 2.2. Data Sources and Variables

Electronic medical records were reviewed to extract demographic information (sex, birthday), clinical diagnoses, vaccination history, visit date, and immunological parameters (T-, B-, NK-cells; IgA/IgG/IgM; C3/C4), which were performed as part of the routine clinical care in the immunization consultation clinic by trained investigators, using predefined data abstraction protocols. To ensure data accuracy and consistency, information from different sources was cross-checked, and discrepancies were resolved through record review and consensus discussion. The following data were obtained through structured caregiver questionnaires, capturing vaccine hesitancy factors, catch-up vaccination status, and reason for no catch-up. Telephone follow-ups were conducted between 1 and 3 months post-vaccination to monitor for any adverse events. Vaccination records were retrieved from the National Immunization Program Information Management System.

The reference of parameters of IgA, IgG, IgM, C3, and C4 were from a multicenter study conducted in 20 medical centers in China, within which eligible healthy children were aged from 0 to 17 years [13]. All comparisons of immunological indices were conducted within age/sex-defined groups, thereby minimizing the confounding effect of age and sex. The reference of parameters of T-cell, B-cell, and NK-cell counts were from a multicenter study conducted in China, including 1075 healthy Chinese children aged from 0 to 18 years [14].

### 2.3. Indicator Definitions

The timely immunization rate for a specific dose was defined as follows: number of timely vaccinated cases at the clinic visit/the number of children who were age-eligible for that dose at the clinic visit ×100% [3]. The definition of timely immunization of different doses of vaccines was illustrated in Table 2, according to the China National Immunization Program Vaccine Immunization Schedule and Instructions for Children [4].

The catch-up vaccination rate is defined as the proportion of children who received the age-appropriate vaccine(s) after the initial consultation visit among all children eligible for catch-up vaccination at the follow-up time point. The eligible case definitions for each vaccine were as follows. For inactivated vaccines, the eligible population was defined as individuals who were recommended to receive inactivated vaccines or were eligible for catch-up vaccination with both inactivated and live attenuated vaccines and who had reached the corresponding vaccination age at the time of follow-up for each NIP vaccine listed in Table 1. For live attenuated vaccines, the eligible population was defined as individuals who were eligible for a catch-up vaccination with both inactivated and live attenuated vaccines and who had reached the corresponding vaccination age at the time of follow-up for each NIP vaccine listed in Table 1.

Adverse events following immunization (AEFI) are defined as any untoward medical occurrences that follow immunization; AEFIs do not necessarily have a causal relationship with the use of a vaccine. A serious AEFI is defined as an event that is life-threatening or results in inpatient hospitalization or prolongation of existing hospitalization, persistent or significant disability/incapacity, a congenital anomaly/birth defect, or death [15].

### 2.4. Statistical Analyses

GraphPad Prism (Version 10.3.0, GraphPad Software, LLC, Boston, MA, USA) was used to perform data analysis and generate graph and figures. The Kolmogorov-Smirnov test was used to test the normality of the data, and numerical data are presented as the mean and SD or median and IQR. ANOVA and the Kruskal–Wallis test were used to compare the differences among disease groups in the following measures: median age at visit; vaccination age at the last dose of NIP vaccines; total T-cell, B-cell, and NK-cell counts; and serum levels of IgA, IgG, IgM, C3, and C4. Chi-square (χ^2^) and Fisher’s exact test were used to compare the differences among disease groups in the timely vaccination rates and catch-up vaccination completion rates. Bonferroni correction was employed for post hoc pairwise comparison. A two-tailed *p* < 0.05 was considered statistically significant. To assess whether the available sample size was sufficient to detect meaningful differences in the primary endpoint, a post hoc power analysis based on the observed effect sizes was conducted, with detailed results provided in the Supplementary Material. Inverse probability weighting was applied to calculate AEFI outcomes.

## 3. Results

### 3.1. Demographic Characteristics and Disease Classification

**Figure 1:**
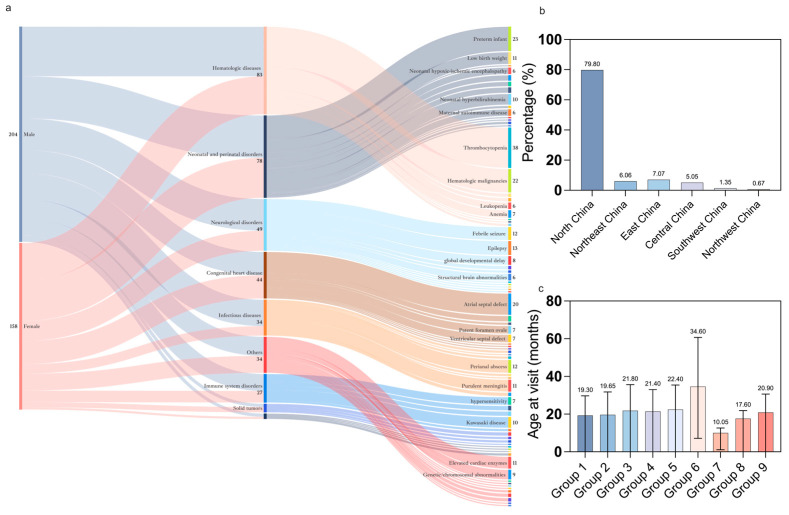
Disease classification and demographic characteristics. (**a**) Spectrum of gender and diseases among included patients. Nine principal categories involving hematologic diseases (24.18%), neonatal/perinatal disorders (21.20%), neurological disorders (13.32%), congenital heart disease (11.96%), infectious diseases (9.24%), immune system disorders (7.34%), solid tumors (2.17%), vaccination adverse effects (1.36%), and others (including genetic/chromosomal abnormalities and some transient conditions, 9.2%) groups. (**b**) Region distribution of included patients. (**c**) Comparison of age at visit across the nine groups. Data are shown as mean/median. Error bars represent standard deviations/quartile.

We included 305 CSHCNs (56.7% boys); the median age at visit was 20.8 months (IQR 8.5–52.1) and this did not vary significantly by disease group (Figure 1c). Most resided in North China (n = 237, 79.8%) (Figure 1b). The spectrum of primary diagnoses comprised nine categories, including hematological diseases (n = 89, 24.2%), neonatal/perinatal disorders (n = 78, 21.2%), neurological disorders (n = 49, 13.3%), congenital heart disease (n = 44, 12.0%), infectious diseases (n = 34, 9.2%), immune system disorders (n = 27, 7.3%), solid tumors (n = 8, 2.2%), vaccination adverse effects (n = 5, 1.4%), and others (including genetic/chromosomal abnormalities and some transient conditions, n = 34, 9.2%) (Figure 1a). The most common diagnosis was thrombocytopenia (n = 44), followed by prematurity (n = 23) and hematological malignancies (n = 22). Detailed data for gender and disease classification are shown in Table S1.

Among caregivers who visited our clinics from 2022 onwards, the primary reasons for delayed vaccination included caregiver concerns about the child’s underlying health conditions (55.8%), refusal/deferral by the POV provider (49.5%), and recommendations for delay from specialists (13.7%).

### 3.2. Timely Vaccination Rates Across Disease Groups

**Figure 2:**
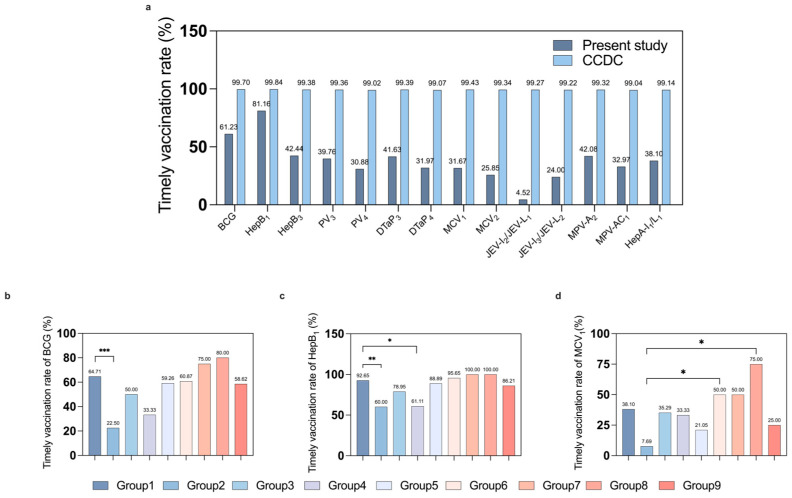
Comparison of timely vaccination rate for each vaccine between the healthy population and across disease groups. (**a**) Comparison of timely vaccination rates: study participants versus 2021 CCDC reported data (each vaccine: *p* < 0.001); timely vaccination rate of BCG (**b**) HepB1 (**c**), and MCV1 (**d**) across the nine groups. Group 1: Hematologic diseases, 2: neonatal/perinatal disorders, 3: neurological disorders, 4: congenital heart disease, 5: infectious diseases, 6: immune system disorders, 7: solid tumors, 8: vaccination adverse effects, and 9: others. * *p* value  <  0.05, ** *p* value  <  0.01, *** *p* value  <  0.001.

Timely vaccination was consistently below the 2021 national benchmarks for healthy children (all *p* < 0.001) with the lowest rates being for the Japanese encephalitis (JE, 24.0%) vaccine and the measles-containing vaccine (MCV, 25.9%). Timely completion rates were 61.2% for BCG, 42.4% for HepB, 32.0% for DTaP, and 30.9% for PV (Figure 2a). Detailed rates for each vaccine dose are shown in Table 2.

The timely vaccination rates for BCG (*p* = 0.001), HepB1 (*p* < 0.001), and MCV1 (*p* = 0.026) were significantly varied among the nine groups, and the Bonferroni correction test showed that the neonatal/perinatal disorders group severely affected the vaccination rates of BCG and the first dose of HepB and MCV. BCG vaccination was administered less frequently to the neonatal/perinatal disorders group compared to the hematological diseases group (Figure 2b). The first HepB dose was significantly less timely in the neonatal/perinatal disorders and congenital heart disease groups than in the hematological diseases group (Figure 2c). The first dose of MCV vaccination rate was significantly lower in the neonatal/perinatal disorders group than in the immune system disorders and vaccination adverse effects groups (Figure 2d). Delayed vaccination with specific vaccines was associated with the type of disease informing us to find out the reasons for delayed vaccination in specific diseases and to make a targeted policy.

### 3.3. Catch-Up Vaccination

**Figure 3:**
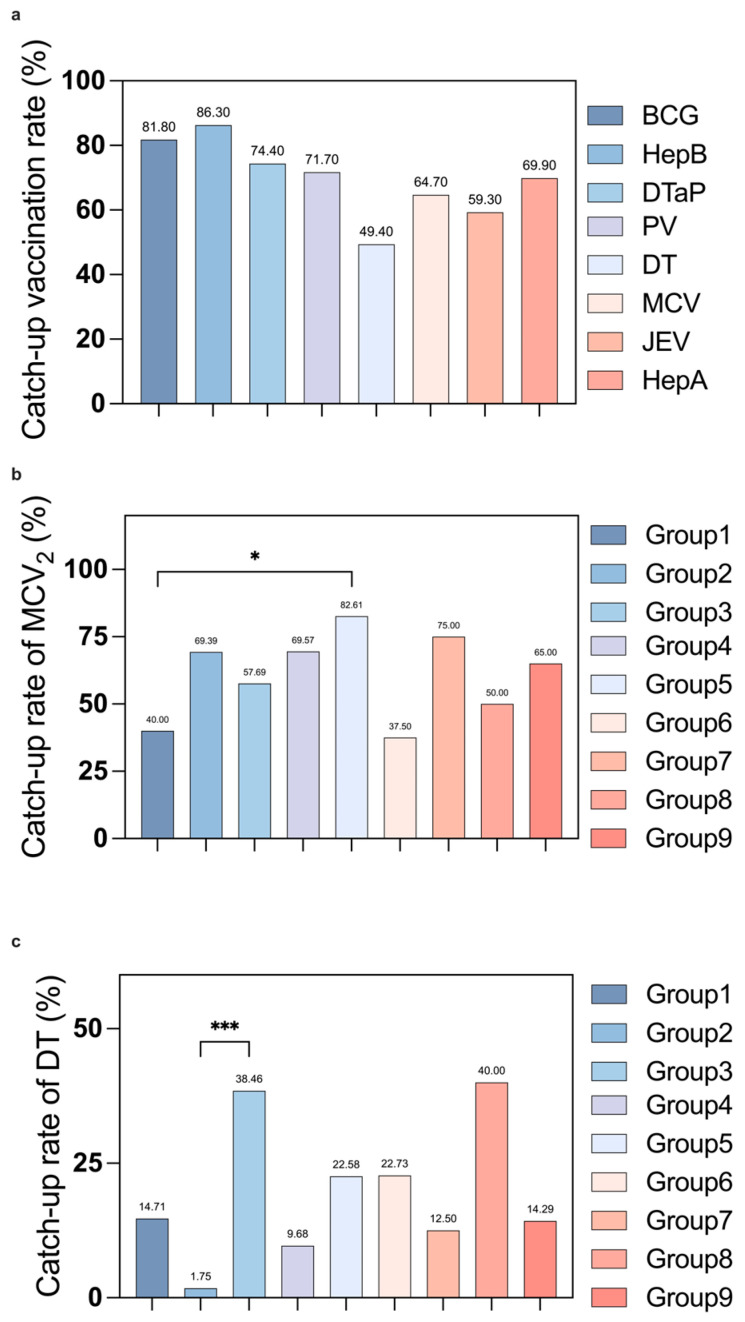
Catch-up vaccination: Overall rate and inter-group comparison across disease groups. (**a**) Despite only BCG and HepB vaccines demonstrating catch-up completion rates above 80%, the overall vaccination coverage was low. (**b**) Catch-up vaccination rate of MCV2 in hematologic diseases group was significantly lower than in infectious diseases (*p* = 0.032). (**c**) Catch-up vaccination rate of DT in neonatal/perinatal disorders group was significantly lower than neurological disorders group (*p* < 0.001). Group 1: Hematologic diseases, 2: neonatal/perinatal disorders, 3: neurological disorders, 4: congenital heart disease, 5: infectious diseases, 6: immune system disorders, 7: solid tumors, 8: vaccination adverse effects, and 9: others. * *p* value  <  0.05, *** *p* value  <  0.001.

Catch-up completion was highest for HepB3 (86.3%) and BCG (81.8%), and lowest for diphtheria and tetanus combined vaccine (DT, 49.4%) (Figure 3a; Table 2). Completion of MCV2 was lower in hematological diseases versus infectious diseases; DT completion was lower in neonatal/perinatal versus neurological disorders (Figure 3b,c). Completion rates for the HepB3, DTaP4, PV4, JE, and hepatitis A vaccines (HepA) were comparable across the nine groups. Despite formal recommendations for the catch-up vaccination, some CSHCNs failed to complete their schedules, exhibiting disparities linked to their underlying conditions. Detailed catch-up completion of other vaccines was shown in Figure 3a. This highlights the need for better mechanisms to enhance collaboration between vaccine counseling clinics and POVs and a deeper investigation into the underlying concerns within specific patient subpopulations to facilitate vaccination tracking.

### 3.4. Cellular, Humoral, and Complement Immunity

**Figure 4:**
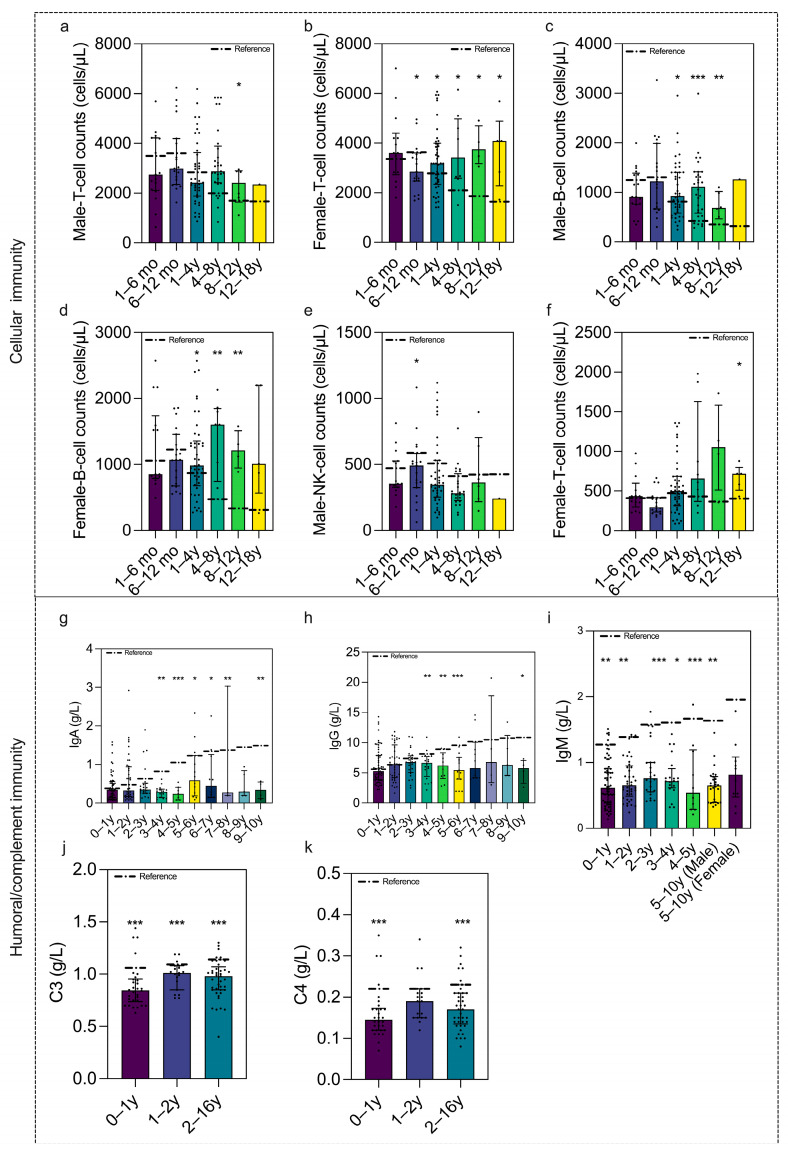
Comparison of cellular, humoral, and complement immunity versus healthy population and across disease groups. (**a**) T–cell counts in males were comparable with the healthy population; (**b**) T–cell counts in females were significantly lower in ages 6–12 months than in the healthy population (*p* = 0.01); B-cell counts were comparable with the healthy population in both males (**c**) and females (**d**); (**e**) NK–cell counts in males were significantly lower in age 6–12 months than in the healthy population (*p* = 0.034). (**f**) NK–cell counts in females were comparable with the healthy population; (**g**) IgA concentrations were significantly lower in age 3–4 y (*p* = 0.0025), 4–5 y (*p* < 0.001), 5–6 y (*p* = 0.05), 6–7 y (0.027), 8–9 y (*p* = 0.003), and 9–10 y (*p* = 0.002) than in the healthy population. (**h**) IgG concentrations were significantly lower in age 3–4 y (*p* = 0.0012), 4–5 y (*p* = 0.004), 5–6 y (*p* < 0.001), and 9–10 y (*p* = 0.01) than in the healthy population. (**i**) IgM concentrations were significantly lower in age 0–1 y (*p* = 0.002), 1–2 y (*p* = 0.0013), 3–4 y (*p* = 0.001), 4–5 y (*p* = 0.048), and 5–10 y (*p* < 0.001 in male, *p* = 0.008 in female) than in the healthy population. (**j**) C3 concentrations were significantly lower in all age groups (*p* < 0.001) than in the healthy population. (**k**) C4 concentrations were significantly lower in age 0–1 y and 2–10 y groups (*p* < 0.001) than in the healthy population. Data are shown as mean/median. Error bars represent standard deviations/quartile (subgroups with KS *p* ≥ 0.05 are presented as mean ± SD, and the error bars indicate SD. Subgroups with KS *p* < 0.05 are presented as median (IQR), and the error bars span the IQR). The corresponding KS *p*-values for each subgroup are provided in the Supplementary Material. Dot–dash line indicates reference value. * *p* value  <  0.05, ** *p* value  <  0.01, and *** *p* value  <  0.001.

**Figure 5:**
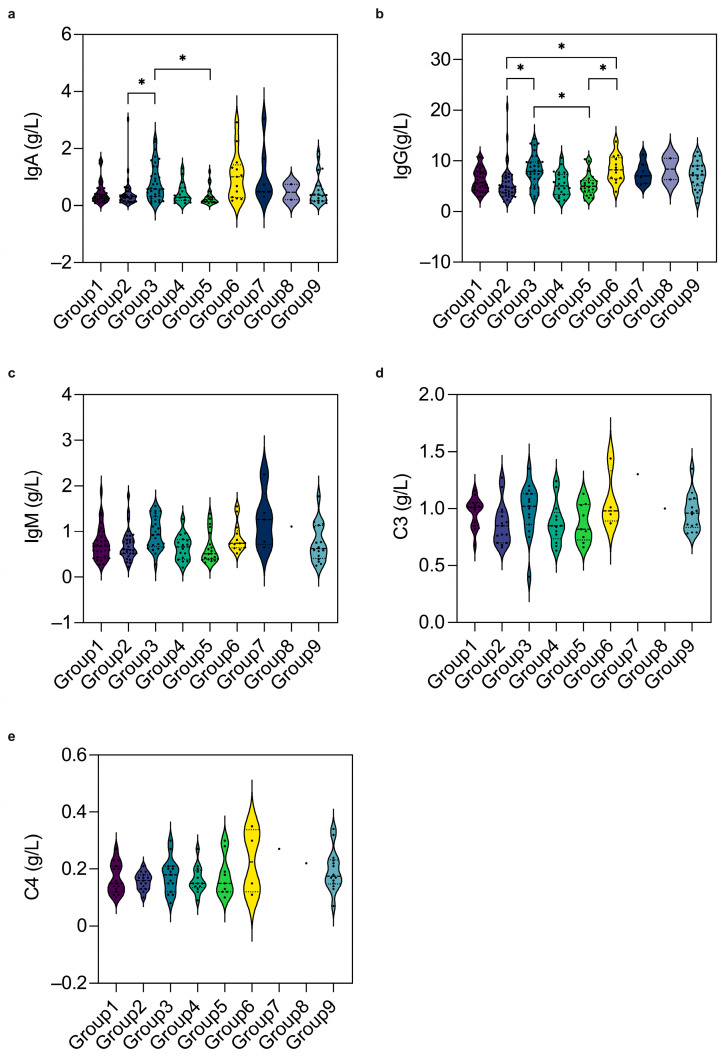
Comparison of humoral and complement immunity across disease groups. (**a**) IgA concentrations in the neonatal/perinatal disorders (*p* = 0.034) and infectious diseases groups (*p* = 0.017) were significantly lower than those in the neurological disorders group. (**b**) IgG concentrations in the neonatal/perinatal disorders group were significantly lower than in the neurological disorders (*p* = 0.011) and immune system disorders groups (*p* = 0.039), and the infectious diseases group was also significantly lower than in the neurological disorders (*p* = 0.043) and immune system disorders groups (*p* = 0.043). Concentrations of IgM (**c**), C3 (**d**), and C4 (**e**) were comparable across the nine groups. Group 1: Hematologic diseases, 2: neonatal/perinatal disorders, 3: neurological disorders, 4: congenital heart disease, 5: infectious diseases, 6: immune system disorders, 7: solid tumors, 8: vaccination adverse effects, and 9: others. Data are shown as mean/median. Error bars represent standard deviations/quartile (subgroups with KS *p* ≥ 0.05 are presented as mean ± SD, and the error bars indicate SD. Sub-groups with KS *p* < 0.05 are presented as median (IQR), and the error bars span the IQR). The corresponding KS *p*-values for each subgroup are provided in the Supplementary Material. * *p* value  <  0.05.

Using age- and sex-stratified evaluations against the corresponding reference ranges across the overall sample, cell counts were largely comparable with the references, except for lower female T-cells and male NK-cells at 6–12 months (Figure 4a–f). Humoral/complement indices were generally lower than reference values, with significantly lower IgA/IgG in neonatal/perinatal and infectious disease groups versus neurological and immune disorder groups (Figure 4g–k and Figure 5a,b) [6]. Concentrations of T-cells, B-cells, NK-cells, IgM, C3, and C4 were comparable across the nine groups. This result underscores a selective humoral immune reduction in specific disease categories, highlighting the need for targeted immunological and vaccination efficacy monitoring in these vulnerable populations.

### 3.5. Adverse Events

Among 164 followed participants, 27 AEFIs were reported, yielding an unweighted AEFI rate of 16.46%, including fever (n = 24), local swelling and redness (n = 2), and rash (n = 1). After applying stabilized inverse probability weights based on the sex, age (months), and disease categories to account for differential responses, the estimated overall AEFI rate was 17.03% (95% CI 11.12–22.86%). No serious AEFIs were reported.

## 4. Discussion

We show systematic shortfalls in timeliness among CSHCNs versus national benchmarks, with early-life delays concentrated in neonatal/perinatal disorders and persistently low catch-up for DT and MCV2. These patterns suggest opportunities for disease-specific guidance and operational fixes at the POV–specialist interface.

Our cohort included children with a wide spectrum of conditions (94 distinct diagnoses). The most prevalent conditions were hematological and neonatal/perinatal disorders, which contrasts with a previous report from Shanghai, China, where neurological disorders were the most common (30.2%) [9]. This discrepancy might be attributable to regional differences or variations in disease classification. Our findings indicate that even for conditions with clear national guidelines permitting routine vaccination—such as stable physiological jaundice, a history of febrile seizures, perianal abscess, and stable congenital heart disease—many children remain under-vaccinated because of caregiver concerns or refusal by POV staff. A previous study found that more than 90% of vaccinators used the immunization schedule and vaccine package inserts to assess contraindications, whereas only 26.24% consulted relevant expert consensus. This highlights an urgent need for enhanced training for POV staff to improve risk assessment, counseling skills, and confidence in vaccine-related decision-making, alongside the development of national disease-specific immunization guidelines to standardize practice; together, these measures may facilitate consistent provider recommendations and help to alleviate caregiver hesitancy [16].

Prior single-center Chinese reports documented very low timeliness among CSHCNs, and international cohorts showed heterogeneity by chronic condition [9,17,18,19]. Our study extends this literature by mapping vaccine-specific delays to disease groups, quantifying post-consultation catch-up completion, and comparing immune indices across groups, highlighting selectively lower humoral immune marker levels in the neonatal/perinatal and infectious disease categories. In our cohort, the timely vaccination rates for HepB1 and BCG were 81.2% and 61.2%, which were considerably higher than before in China [13]. However, we observed that neonatal/perinatal conditions are strongly associated with hesitancy towards BCG and HepB1 vaccinations. To minimize delays and ensure timely BCG and HepB1 catch-up for eligible infants, neonatologists should promptly assess vaccination eligibility or refer these patients to specialized immunization clinics.

Previous studies on vaccination among CSHCNs have primarily focused on hesitancy before medical consultation, with little attention given to catch-up vaccination patterns. Catch-up vaccination completion rates for DT, JE, MCV, and HepA were relatively lower among all NIP vaccines. These differences may reflect a combination of vaccine-specific characteristics and policy-related factors. First, as MCV is a live attenuated vaccine, its use is often contraindicated in individuals with certain health conditions, such as immunodeficiency. In this study, the catch-up rate of MCV in the hematological diseases group was especially low. This observation highlights the need for further research to evaluate the safety and effectiveness of vaccines in these patients, especially hematologic malignancies, with the aim of identifying the optimal criteria and timing for catch-up vaccination. Second, although both JE and HepA are available in live attenuated and inactivated formulations, the inactivated versions—which are safer alternatives for vulnerable populations—are less commonly used in many regions of China and may require out-of-pocket payment. Differences in vaccine availability, formulation preference, and cost may be associated with lower completion rates of catch-up vaccination for JE, MCV, and HepA. The recommended vaccination age for the DT vaccine is six years old, which coincides with a child’s transition from kindergarten to primary school in China. The notably low catch-up vaccination rate among this special population is likely attributable to oversight during this critical transitional period, where healthcare management responsibilities are transferred [20]. It is imperative to strengthen vaccination supervision and management for these vulnerable children during this phase. These interpretations are exploratory in nature and would require further thematic analysis of questionnaire data on vaccination hesitancy for validation.

A novel aspect of this study is its analysis of catch-up rates for specific vaccines following specialist consultation and its comparison of these rates across disease groups, yielding insights to inform targeted vaccination strategies. This study underscores the need for effective educational strategies for caregivers regarding the importance and safety of vaccination. Although POV staff have the most frequent contact with parents, they are often disconnected from specialized vaccine consultation clinics. This lack of integration means they must rely on information relayed by caregivers, which can hinder their ability to provide effective and consistent education. Establishing a direct communication channel between specialist clinics and POVs could substantially enhance this educational role.

Our study unveils a novel and critical finding that CSHCNs in the neonatal/perinatal disorder and infectious disease groups had significantly lower levels of humoral and complement immune function. The neonatal/perinatal periods are critical for immune system maturation, and significant physiological stress or inflammation during this time can impede normal immune development. However, the causal directionality between lower humoral immune levels and vaccination delays remains unclear in this population: lower IgA/IgG may prompt clinical vaccination deferral due to concerns about insufficient immune response, while vaccination delays may also lead to impaired humoral immune maturation due to inadequate antigen stimulation, a temporal relationship that cannot be determined by the cross-sectional design of this study. Comprehensive immune function assessment is therefore essential for individualized risk–benefit analysis before vaccination. Given the uncertain efficacy of immunization in this population, further longitudinal studies were needed to address this critical matter to make a tailored vaccination protocol for this group, which may include rigorous post-vaccination serological testing, adjusted schedules, or even specialized formulations—guided by immune profiling. However, it is safe for catch-up vaccination in this group.

However, this study has certain limitations. First, as a single-center analysis with participants drawn primarily from northern China, the generalizability of our findings to all children with special healthcare needs may be limited. Second, exclusions related to incomplete medical records may have introduced selection bias, as children with missing diagnostic or vaccination documentation could differ systematically from those included in the analysis. Third, follow-up for adverse events following immunization was incomplete, which may have led to an underestimation of mild adverse events. Although no serious AEFIs were observed, this limitation should be considered when interpreting safe-ty-related findings. Finally, the relatively small sample size in some disease subgroups limited statistical power for between-group comparisons, and the observational design precludes causal inference regarding factors influencing vaccination delay, catch-up completion, or immune profiles. Future multi-center studies with broader geographic coverage, larger sample sizes, and longitudinal follow-up are warranted to further validate and extend our findings.

In summary, based on the findings of this study, we propose the following three key policy recommendations:

1. Develop national, disease-specific guidelines and decision aids for POV staff for BCG/HepB1 in neonatal/perinatal disorders and for MCV/JE/HepA formulations in immunologically vulnerable children and implement guideline-based training for POV providers through the tiered healthcare system to strengthen their vaccination decision-making capacity and reduce unnecessary referrals.
2. Formalize referral–feedback mechanisms between specialist consultation clinics and POVs services by implementing shared eligibility notes and proactive recall for DT vaccination at school entry; such a referral–feedback loop can bridge consultation-clinic workflows with community vaccination services, enabling consultation-based eligibility assessments to be transmitted to community clinics and thereby supporting timely implementation of catch-up recommendations. In areas where digital integration is limited, periodic reconciliation provides a low-cost alternative. A tiered rollout—from standardized documentation to electronic interoperability—may maximize scalability and equity.
3. Integrating targeted immunological monitoring may support individualized catch-up planning for clinically complex CSHCNs. Future prospective studies with post-vaccination serology and longitudinal follow-up are needed to test the working hypothesis that baseline immune vulnerability influences catch-up decision-making and vaccine responses after catch-up.

## 5. Conclusions

Children with special healthcare needs (CSHCNs) in China experience substantial delays in routine immunization, with timeliness varying significantly according to the specific vaccine and the child’s underlying medical condition. Improving protection for CSHCNs will require disease-aware timeliness targets, vaccine-formulation choices aligned to risk, and tighter POV–specialist integration—priorities that are actionable within China’s NIP.

## Figures and Tables

**Table 1:** Immunization schedules and instructions for vaccines of the national immunization program (2021 version) in China [1].

Vaccines	Birth	1 mo	2 mos	3 mos	4 mos	5 mos	6 mos	8 mos	9 mos	18 mos	2 yrs	3 yrs	4 yrs	5 yrs	6 yrs
HepB	1	2					3								
BCG	1														
PV			1	2	3	4									
DTaP				1	2	3				4					
DT															5
MCV								1		2					
JE-L								1			2				
JE-I								1/2			3				
MPSV-A							1		2						
MPSV-AC												3			4
HepA-L										1					
HepA-I										1	2				

Note: BCG, Bacille Calmette–Guérin vaccine; PV, poliomyelitis vaccine; DTaP, diphtheria–tetanus–acellular pertussis vaccine; DT, diphtheria–tetanus vaccine; MCV, measles-containing vaccine; JE-L, live attenuated Japanese encephalitis vaccine; JE-I, inactivated Japanese encephalitis vaccine; MPSV-A, group A meningococcal polysaccharide vaccine; MPSV-AC, group A and C meningococcal polysaccharide vaccine; HepA-L, live attenuated hepatitis A vaccine; HepA-I, inactivated hepatitis A vaccine; and HepB, hepatitis B vaccine. Abbreviations: mo = month; yr = year.

**Table 2:** Timely immunization rate and catch-up vaccination rate of each vaccine.

	Definition of Timely Immunization	Timely Immunization Rate (*n*/N, %)	Catch-Up Vaccination Rate (*n*/N, %)
BCG	Within 24 h	169/276, 61.23	54/66, 81.82
HepB_1_	Within 3 months	224/276, 81.16	25/26, 96.15
HepB_3_	Within 12 months	101/238, 42.44	107/124, 86.29
PV_3_	Within 12 months	99/249, 39.76	112/141, 79.43
PV_4_	Within 5 years	21/68, 30.88	104/145, 71.72
DTaP_3_	Within 12 months	102/245, 41.63	110/133, 82.71
DTaP_4_	Within 24 months	47/147, 31.97	122/164, 74.39
MCV_1_	Within 12 months	70/221, 31.67	89/124, 71.77
MCV_2_	Within 24 months	38/147, 25.85	88/136, 64.71
JE-I_2_/JE-L_1_	Within 12 months	10/221, 4.52	136/191, 71.20
JE-I_3_/JE-L_2_	Within 36 months	30/125, 24.00	108/182, 59.34
MPSV-A_2_	Within 18 months	85/202, 42.08	NA
MPSV-AC_1_	Within 4 years	30/91, 32.97	NA
HepA-I_1_/HepA-L_1_	Within 24 months	56/147, 38.10	102/146, 69.86
HepA-I_2_	Within 36 months	33/125, 26.40	NA

Note: BCG, Bbacille Calmette–Guérin vaccine; HepB, hepatitis B vaccine; PV, poliomyelitis vaccine; DTaP, diphtheria–tetanus–acellular pertussis vaccine; MCV, mea-sles-containing vaccine; JE-L, live attenuated Japanese encephalitis vaccine; JE-I, inactivated Japa-nese encephalitis vaccine; MPSV-A, group A meningococcal polysaccharide vaccine; MPSV-AC, group A and C meningococcal polysaccharide vaccine; HepA-I, inactivated hepatitis A vaccine; and HepA-L, live attenuated hepatitis A vaccine.

## Data Availability

All data generated or analyzed during this study are included in this published article and its Supplementary Material.

## Supplementary Materials

The following supporting information can be downloaded at: https://www.mdpi.com/article/10.3390/vaccines14020149/s1, Table S1. Gender and disease classification. Table S2. Vaccination coverage of National Immunization Program (NIP) vaccines by vaccine type in 2019–2021. Table S3. Catch-up Eligibility Windows for Missed Early-life Doses of National Immunization Program (2021 Guidance). Table S4 (1). Summary of Post-hoc Statistical Power for 9 Groups’ Primary Endpoint: timely vaccination of BCG. Table S4 (2). Summary of Post-hoc Statistical Power for 9 Groups’ Primary Endpoint: timely vaccination of HepB1. Table S4 (3). Summary of Post-hoc Statistical Power for 9 Groups’ Primary Endpoint: timely vaccination of MCV1. Table S5. Cellular immune indices (absolute counts) by age and sex group. Table S6. Humoral immune indices and complement components by age and sex group. Table S7 (1). C3 descriptive statistics by group. Table S7 (2). Pairwise post-hoc comparisons for C3 (Bonferroni-adjusted) with effect sizes. Table S8 (1). C4 descriptive statistics by group. Table S8 (2). Pairwise post-hoc comparisons for C4 (Bonferroni-adjusted) with effect sizes. Table S9 (1). IgA descriptive statistics by group. Table S9 (2). Pairwise post-hoc comparisons for IgA (Bonferroni-adjusted) with effect sizes. Table S10 (1). IgG descriptive statistics by group. Table S10 (2). Pairwise post-hoc comparisons for IgG (Bonferroni-adjusted) with effect sizes. Table S11 (1). IgM descriptive statistics by group. Table S11 (2). Pairwise post-hoc comparisons for IgM (Bonferroni-adjusted) with effect sizes. Figure S1. Study flow diagram. Figure S2. Vaccination coverage of National Immunization Program (NIP) vaccines by vaccine type in 2019–2021. Figure S3. Normal Q–Q plots for immunological indicators across subgroups. Figure S4. Normal Q–Q plots of humoral and complement immune indicators across study groups.
