# Supplementary material for: Timeliness of Routine Vaccination, Catch-Up Completion, and Immune Function in Chinese Children with Special Healthcare Needs: A Retrospective Cohort Study
Source: Vaccines (Basel). 2026 Jan 31;14(2):149. doi: 10.3390/vaccines14020149 (PMC12945083; doi:10.3390/vaccines14020149)
Supplement: Supplementary file 1 [file vaccines-14-00149-s001.zip › vaccines-4107110-supplementary.pdf]

Timeliness of Routine Vaccination, Catch-up Completion, and Immune Function in Chinese Children with Special Healthcare Needs in China: A Retrospective Cohort Study

SUPPLEMENTARY MATERIALS

Table S1. Gender and disease classification.

Table S2. Vaccination coverage of National Immunization Program (NIP) vaccines by vaccine type in 2019–2021.

Table S3. Catch-up Eligibility Windows for Missed Early-life Doses of National Immunization Program (2021 Guidance).

Table S4 (1). Summary of Post-hoc Statistical Power for 9 Groups' Primary Endpoint: timely vaccination of BCG.

Table S4 (2). Summary of Post-hoc Statistical Power for 9 Groups' Primary Endpoint: timely vaccination of HepB1.

Table S4 (3). Summary of Post-hoc Statistical Power for 9 Groups' Primary Endpoint: timely vaccination of MCV1.

Table S5. Cellular immune indices (absolute counts) by age and sex group.

Table S6. Humoral immune indices and complement components by age and sex group.

Table S7 (1). C3 descriptive statistics by group.

Table S7 (2). Pairwise post-hoc comparisons for C3 (Bonferroni-adjusted) with effect sizes.

Table S8 (1). C4 descriptive statistics by group.

Table S8 (2). Pairwise post-hoc comparisons for C4 (Bonferroni-adjusted) with effect sizes.

Table S9 (1). IgA descriptive statistics by group.

Table S9 (2). Pairwise post-hoc comparisons for IgA (Bonferroni-adjusted) with effect sizes.

Table S10 (1). IgG descriptive statistics by group.

Table S10 (2). Pairwise post-hoc comparisons for IgG (Bonferroni-adjusted) with effect sizes.

Table S11 (1). IgM descriptive statistics by group.

Table S11 (2). Pairwise post-hoc comparisons for IgM (Bonferroni-adjusted) with effect sizes.

Figure S1. Study flow diagram.

Figure S2. Vaccination coverage of National Immunization Program (NIP) vaccines by vaccine type in 2019–2021.

Figure S3. Normal Q–Q plots for immunological indicators across subgroups.

Figure S4. Normal Q–Q plots of humoral and complement immune indicators across study groups.

Table S1. Gender and disease classification

Gender/Disease classification	Specific diseases	N
Male	Hematologic diseases	47
Male	Neonatal and perinatal disorders	40
Male	Neurological disorders	30
Male	Congenital heart disease	22
Male	Infectious diseases	25
Male	Others	18
Male	Immune system disorders	16
Male	Solid tumors	3
Male	Vaccination adverse effects	3
Female	Hematologic diseases	36
Female	Neonatal and perinatal disorders	38
Female	Neurological disorders	19
Female	Congenital heart disease	22
Female	Infectious diseases	9
Female	Others	16
Female	Immune system disorders	11
Female	Solid tumors	5
Female	Vaccination adverse effects	2
Neonatal and perinatal disorders	Preterm infant	23
Neonatal and perinatal disorders	Low birth weight	11
Neonatal and perinatal disorders	Short Bowel Syndrome	1
Neonatal and perinatal disorders	Neonatal hypoxic-ischemic encephalopathy	6
Neonatal and perinatal disorders	Biliary atresia	5
Neonatal and perinatal disorders	Necrotizing enterocolitis	4
Neonatal and perinatal disorders	Neonatal respiratory distress syndrome	5
Neonatal and perinatal disorders	Neonatal hyperbilirubinemia	10
Neonatal and perinatal disorders	Cholestasis	2
Neonatal and perinatal disorders	Maternal autoimmune disease	6
Neonatal and perinatal disorders	Abnormal amniocentesis results	1
Neonatal and perinatal disorders	Bronchopulmonary dysplasia	1
Neonatal and perinatal disorders	Neonatal sepsis	2
Neonatal and perinatal disorders	Congenital CMV infection	1
Hematologic diseases	Thrombocytopenia	38
Hematologic diseases	Hematologic malignancies	22
Hematologic diseases	Leukocytosis	3
Hematologic diseases	Hereditary spherocytosis	3
Hematologic diseases	Leukopenia	6
Hematologic diseases	Anemia	7
Hematologic diseases	Pancytopenia	1

Hematologic diseases	Hemophilia	1
Hematologic diseases	Thrombocytosis	2
Neurological disorders	Febrile seizure	12
Neurological disorders	Epilepsy	13
Neurological disorders	global developmental delay	8
Neurological disorders	Afebrile seizure	3
Neurological disorders	Hydrocephalus	2
Neurological disorders	Structural brain abnormalities	6
Neurological disorders	Limb tremor	1
Neurological disorders	Cerebellar ataxia	1
Neurological disorders	Acute necrotizing encephalopathy	1
Neurological disorders	Progressive muscular dystrophy	1
Neurological disorders	Behavioral abnormalities	1
Congenital heart disease	Atrial septal defect	20
Congenital heart disease	Patent ductus arteriosus	5
Congenital heart disease	Transposition of great arteries	2
Congenital heart disease	Patent foramen ovale	7
Congenital heart disease	Ventricular septal defect	7
Congenital heart disease	Tetralogy of Fallot	1
Congenital heart disease	Coarctation of aorta	1
Congenital heart disease	Coronary-pulmonary artery fistula	1
Infectious diseases	Clostridioides difficile infection	2
Infectious diseases	Bacterial enteritis	1
Infectious diseases	Severe pneumonia	2
Infectious diseases	Perianal abscess	12
Infectious diseases	Oral thrush	2
Infectious diseases	Recurrent infections	1
Infectious diseases	Purulent meningitis	11
Infectious diseases	Urinary tract infection	3
Immune system disorders	hypersensitivity	7
Immune system disorders	Anaphylaxis	4
Immune system disorders	IgA Vasculitis	4
Immune system disorders	Kawasaki disease	10
Immune system disorders	Inflammatory bowel disease	2
Solid tumors	Hepatoblastoma	3
Solid tumors	Retinoblastoma	2

Solid tumors	Nephroblastoma	2
Solid tumors	Rhabdomyosarcoma	1
Vaccination adverse effects	Vaccination site induration	1
Vaccination adverse effects	Dyspnea	1
Vaccination adverse effects	Axillary lymphadenopathy	1
Vaccination adverse effects	Fever	2
Others	Elevated cardiac enzymes	11
Others	Genetic/chromosomal abnormalities	9
Others	Cleft palate	1
Others	Hyperglycemia	1
Others	Hypothyroidism	1
Others	Hypoglycemia	1
Others	Recurrent hematochezia	2
Others	Lymphatic Malformations	3
Others	Abnormal liver function	3
Others	Choledochalcyst	1
Others	Renal tubular acidosis	1

Table S2. Vaccination coverage of National Immunization Program (NIP) vaccines by vaccine type in 2019–2021

Vaccines	2019 (%)	2020 (%)	2021 (%)
HepB1	99.85	99.84	99.84
HepB2	99.62	99.62	99.64
HepB3	99.41	99.38	99.38
BCG	99.68	99.67	99.68
PV1	99.48	99.42	99.43
PV2	99.53	99.44	99.47
PV3	99.47	99.34	99.36
PV4	99.03	98.98	99.02
DTaP1	99.18	99.4	99.39
DTaP2	99.16	99.44	99.44
DTaP3	99.05	99.41	99.39
DTaP4	98.81	99.01	99.06
DT	98.91	98.69	98.71
MCV1	98.83	99.22	99.43
MCV2	98.47	99.04	99.34
MPV-A1	99.19	98.65	99.26
MPV-A2	99.27	98.59	99.32
MPV-AC1	98.97	99.16	99.04
MPV-AC2	98.89	99	98.93
JEV-L1	99.19	99.25	99.27
JEV-L2	99.21	99.17	99.22
HepA1	97.87	99.1	99.14

Table S3. Catch-up Eligibility Windows for Missed Early-life Doses of National Immunization Program (2021 Guidance)

Vaccine (example early-life dose)	Catch-up eligibility window / age threshold
Hepatitis B (HepB1/HepB series)	If any doses are missing, administer the remaining dose(s) as soon as possible during childhood (no special upper age limit specified in the guidance).
BCG	<3 months: can be given directly; 3 months to <4 years: eligible if tuberculin (PPD) test is negative; ≥4 years: not recommended for catch-up.
PV	<4 years: catch up to a total of 3 doses; ≥4 years: catch up to a total of 4 doses.
DTaP / DT	Ages 3 months to <6 years: catch up missing DTaP doses; ≥6 years: follow DT catch-up rules to complete required doses.
MCV	Children missing required dose(s) are eligible for catch-up during childhood; complete the remaining dose(s).
JE-L	If JE vaccine has not been received, eligible to complete a 2-dose JE-L series.
JE-I	If JE vaccine has not been received, eligible to complete a 4-dose JE-I series.
MPSV-A / MPSV-AC	<24 months: eligible to catch up missing MPSV-A doses; ≥24 months: MPSV-A is generally not used for catch-up—complete MPSV-AC as scheduled.
HepA-L / HepA-I	If not vaccinated, eligible to complete either 1-dose HepA-L or 2-dose HepA-I schedule.

Note: This table summarizes catch-up eligibility windows and key conditions from China’s National Immunisation Program (NIP) immunization schedule and catch-up guidance (2021 version).

Table S4 (1). Summary of Post-hoc Statistical Power for 9 Groups' Primary Endpoint: timely vaccination of BCG

Group 1	Group 2	**n1**	**n2**	**p1**	**p2**	**Cohen_h**	**Power**
1	2	68	40	0.647	0.225	0.881	0.993
1	3	68	38	0.647	0.5	0.299	0.314
1	4	68	18	0.647	0.333	0.638	0.673
1	5	68	27	0.647	0.593	0.112	0.078
1	6	68	29	0.647	0.586	0.125	0.087
1	7	68	23	0.647	0.609	0.079	0.062
1	8	68	8	0.647	0.75	-0.225	0.092
1	9	68	5	0.647	0.8	-0.345	0.116
2	3	40	38	0.225	0.5	-0.582	0.729
2	4	40	18	0.225	0.333	-0.243	0.137
2	5	40	27	0.225	0.593	-0.769	0.87
2	6	40	29	0.225	0.586	-0.756	0.873
2	7	40	23	0.225	0.609	-0.802	0.865
2	8	40	8	0.225	0.75	-1.106	0.815
2	9	40	5	0.225	0.8	-1.226	0.734
3	4	38	18	0.5	0.333	0.34	0.221
3	5	38	27	0.5	0.593	-0.186	0.115
3	6	38	29	0.5	0.586	-0.173	0.108
3	7	38	23	0.5	0.609	-0.219	0.132
3	8	38	8	0.5	0.75	-0.524	0.27
3	9	38	5	0.5	0.8	-0.644	0.272
4	5	18	27	0.333	0.593	-0.526	0.409
4	6	18	29	0.333	0.586	-0.513	0.401
4	7	18	23	0.333	0.609	-0.559	0.427
4	8	18	8	0.333	0.75	-0.863	0.529
4	9	18	5	0.333	0.8	-0.983	0.494
5	6	27	29	0.593	0.586	0.013	0.05
5	7	27	23	0.593	0.609	-0.033	0.052
5	8	27	8	0.593	0.75	-0.337	0.134
5	9	27	5	0.593	0.8	-0.457	0.156
6	7	29	23	0.586	0.609	-0.046	0.053
6	8	29	8	0.586	0.75	-0.35	0.142
6	9	29	5	0.586	0.8	-0.47	0.163
7	8	23	8	0.609	0.75	-0.304	0.115
7	9	23	5	0.609	0.8	-0.424	0.138
8	9	8	5	0.75	0.8	-0.12	0.055

Table S4 (2). Summary of Post-hoc Statistical Power for 9 Groups' Primary Endpoint: timely vaccination of HepB1

Group 1	Group 2	**n1**	**n2**	**p1**	**p2**	**Cohen_h**	**Power**
1	2	68	40	0.926	0.6	0.82	0.984
1	3	68	38	0.926	0.789	0.404	0.514
1	4	68	18	0.926	0.611	0.798	0.853
1	5	68	27	0.926	0.889	0.13	0.088
1	6	68	29	0.926	0.862	0.212	0.159
1	7	68	23	0.926	0.957	-0.129	0.083
1	8	68	8	0.926	1	-0.549	0.312
1	9	68	5	0.926	1	-0.549	0.22
2	3	40	38	0.6	0.789	-0.416	0.451
2	4	40	18	0.6	0.611	-0.023	0.051
2	5	40	27	0.6	0.889	-0.69	0.791
2	6	40	29	0.6	0.862	-0.608	0.704
2	7	40	23	0.6	0.957	-0.949	0.952
2	8	40	8	0.6	1	-1.369	0.942
2	9	40	5	0.6	1	-1.369	0.823
3	4	38	18	0.789	0.611	0.393	0.28
3	5	38	27	0.789	0.889	-0.274	0.193
3	6	38	29	0.789	0.862	-0.192	0.122
3	7	38	23	0.789	0.957	-0.533	0.523
3	8	38	8	0.789	1	-0.953	0.688
3	9	38	5	0.789	1	-0.953	0.518
4	5	18	27	0.611	0.889	-0.667	0.592
4	6	18	29	0.611	0.862	-0.586	0.497
4	7	18	23	0.611	0.957	-0.927	0.838
4	8	18	8	0.611	1	-1.347	0.887
4	9	18	5	0.611	1	-1.347	0.759
5	6	27	29	0.889	0.862	0.081	0.061
5	7	27	23	0.889	0.957	-0.26	0.15
5	8	27	8	0.889	1	-0.68	0.393
5	9	27	5	0.889	1	-0.68	0.287
6	7	29	23	0.862	0.957	-0.341	0.231
6	8	29	8	0.862	1	-0.761	0.478
6	9	29	5	0.862	1	-0.761	0.349
7	8	23	8	0.957	1	-0.42	0.176
7	9	23	5	0.957	1	-0.42	0.136
8	9	8	5	1	1	0	0.05

Table S4 (3). Summary of Post-hoc Statistical Power for 9 Groups' Primary Endpoint: timely vaccination of MCV1

Group 1	Group 2	**n1**	**n2**	**p1**	**p2**	**Cohen_h**	**Power**
1	2	68	40	0.381	0.077	0.768	0.971
1	3	68	38	0.381	0.353	0.058	0.059
1	4	68	18	0.381	0.333	0.099	0.066
1	5	68	27	0.381	0.211	0.377	0.381
1	6	68	29	0.381	0.25	0.283	0.248
1	7	68	23	0.381	0.5	-0.24	0.169
1	8	68	8	0.381	0.5	-0.24	0.099
1	9	68	5	0.381	0.75	-0.764	0.378
2	3	40	38	0.077	0.353	-0.71	0.88
2	4	40	18	0.077	0.333	-0.669	0.654
2	5	40	27	0.077	0.211	-0.391	0.349
2	6	40	29	0.077	0.25	-0.485	0.512
2	7	40	23	0.077	0.5	-1.009	0.971
2	8	40	8	0.077	0.5	-1.009	0.74
2	9	40	5	0.077	0.75	-1.532	0.898
3	4	38	18	0.353	0.333	0.041	0.052
3	5	38	27	0.353	0.211	0.319	0.245
3	6	38	29	0.353	0.25	0.225	0.15
3	7	38	23	0.353	0.5	-0.299	0.204
3	8	38	8	0.353	0.5	-0.299	0.12
3	9	38	5	0.353	0.75	-0.822	0.408
4	5	18	27	0.333	0.211	0.278	0.149
4	6	18	29	0.333	0.25	0.184	0.094
4	7	18	23	0.333	0.5	-0.34	0.191
4	8	18	8	0.333	0.5	-0.34	0.126
4	9	18	5	0.333	0.75	-0.863	0.401
5	6	27	29	0.211	0.25	-0.094	0.064
5	7	27	23	0.211	0.5	-0.617	0.586
5	8	27	8	0.211	0.5	-0.617	0.335
5	9	27	5	0.211	0.75	-1.141	0.649
6	7	29	23	0.25	0.5	-0.524	0.466
6	8	29	8	0.25	0.5	-0.524	0.259
6	9	29	5	0.25	0.75	-1.047	0.58
7	8	23	8	0.5	0.5	0	0.05
7	9	23	5	0.5	0.75	-0.524	0.186
8	9	8	5	0.5	0.75	-0.524	0.151

Table S5. Cellular immune indices (absolute counts) by age and sex group.

Indicator	Age group (months)	Sex	n	Normality p (Shapiro-Wilk)	Summary statistic	Reference mean	Test	p value	Significant vs reference*	Difference vs reference	Effect size
T	1-6	Male	17	0.6660 (Normal)	3029.9 ± 1342.6	3488	One-sample t-test	0.1786	No	-458.08	g=-0.32
T	1-6	Female	14	0.4737 (Normal)	3786.2 ± 1430.3	3369	One-sample t-test	0.2949	No	417.18	g=0.27
T	6-12	Male	19	0.0313 (Non-normal)	2989.0 (IQR 1655.8)	3595	Wilcoxon signed-rank	0.4653	No	-357.39	r_rb=-0.20
T	6-12	Female	19	0.2703 (Normal)	3005.0 ± 938.2	3625	One-sample t-test	0.0100	Yes	-620.00	g=-0.63
T	12-48	Male	41	0.0277 (Non-normal)	2424.2 (IQR 1611.1)	2843	Wilcoxon signed-rank	0.4720	No	-136.23	r_rb=-0.13
T	12-48	Female	42	0.0269 (Non-normal)	3177.9 (IQR 1584.9)	2778	Wilcoxon signed-rank	0.0218	Yes	473.02	r_rb=0.41
T	48-96	Male	30	0.0001 (Non-normal)	2979.6 (IQR 1464.4)	1989	Wilcoxon signed-rank	<0.0001	Yes	1096.83	r_rb=0.86
T	48-96	Female	8	0.6681 (Normal)	3675.2 ± 1571.1	2092	One-sample t-test	0.0247	Yes	1583.16	g=0.88
T	96-144	Male	6	0.2190 (Normal)	2266.1 ± 748.9	1686	One-sample t-test	0.1162	No	580.12	g=0.62

T	96-144	Female	4	0.8336 (Normal)	3873.9 ± 799.6	1864	One-sample t-test	0.0152	Yes	2009.89	g=1.44
T	>144	Male	1	NA (Non-normal)	2348.0 (IQR 0.0)	1661	Wilcoxon signed-rank	1.0000	No	687.02	r_rb=NA
T	>144	Female	5	0.8672 (Normal)	3686.2 ± 1491.5	1617	One-sample t-test	0.0361	Yes	2069.24	g=1.01
B	1-6	Male	17	0.6269 (Normal)	1039.3 ± 451.6	1234	One-sample t-test	0.0945	No	-194.69	g=-0.41
B	1-6	Female	14	0.0236 (Non-normal)	850.5 (IQR 775.0)	1058	Wilcoxon signed-rank	0.3964	No	127.94	r_rb=0.26
B	6-12	Male	19	0.1536 (Normal)	1317.5 ± 750.5	1302	One-sample t-test	0.9293	No	15.48	g=0.02
B	6-12	Female	19	0.0946 (Normal)	1092.8 ± 438.8	1226	One-sample t-test	0.2023	No	-133.22	g=-0.29
B	12-48	Male	41	0.0107 (Non-normal)	925.5 (IQR 799.8)	822	Wilcoxon signed-rank	0.0263	Yes	190.81	r_rb=0.40
B	12-48	Female	42	0.0297 (Non-normal)	983.5 (IQR 626.3)	867	Wilcoxon signed-rank	0.0310	Yes	202.01	r_rb=0.38
B	48-96	Male	30	0.0165 (Non-normal)	1113.3 (IQR 787.8)	423	Wilcoxon signed-rank	<0.0001	Yes	604.90	r_rb=0.92
B	48-96	Female	8	0.3349 (Normal)	1394.6 ± 604.2	473	One-sample t-test	0.0035	Yes	921.61	g=1.33
B	96-144	Male	6	0.7945 (Normal)	719.7 ± 284.0	350	One-sample t-test	0.0243	Yes	369.66	g=1.04

B	96-144	Female	4	0.9925 (Normal)	1223.9 ± 292.2	333	One-sample t-test	0.0089	Yes	890.91	g=1.74
B	>144	Male	1	NA (Non-normal)	1260.8 (IQR 0.0)	316	Wilcoxon signed-rank	1.0000	No	944.83	r_rb=NA
B	>144	Female	5	0.2774 (Normal)	1307.3 ± 859.7	309	One-sample t-test	0.0603	No	998.32	g=0.84
NK	1-6	Male	17	0.0002 (Non-normal)	375.0 (IQR 262.2)	471	Wilcoxon signed-rank	0.9632	No	-42.45	r_rb=-0.30
NK	1-6	Female	14	0.0001 (Non-normal)	436.9 (IQR 286.8)	411	Wilcoxon signed-rank	0.5095	No	25.94	r_rb=0.08
NK	6-12	Male	19	0.5222 (Normal)	465.5 ± 233.2	588	One-sample t-test	0.0343	Yes	-122.48	g=-0.50
NK	6-12	Female	19	0.0007 (Non-normal)	308.9 (IQR 175.7)	416	Wilcoxon signed-rank	0.2432	No	-86.09	r_rb=-0.45
NK	12-48	Male	41	0.0002 (Non-normal)	346.4 (IQR 272.0)	508	Wilcoxon signed-rank	0.0330	Yes	-129.84	r_rb=-0.38
NK	12-48	Female	42	0.0030 (Non-normal)	498.5 (IQR 358.2)	473	Wilcoxon signed-rank	0.3202	No	52.29	r_rb=0.18
NK	48-96	Male	30	<0.0001 (Non-normal)	312.5 (IQR 243.3)	406	Wilcoxon signed-rank	0.6883	No	-104.84	r_rb=-0.68
NK	48-96	Female	8	0.0552 (Normal)	889.7 ± 674.2	432	One-sample t-test	0.0963	No	457.71	g=0.59
NK	96-144	Male	6	0.5413 (Normal)	441.8 ± 277.9	423	One-sample t-test	0.8748	No	18.82	g=0.05

NK	96-144	Female	4	0.9370 (Normal)	1049.1 ± 565.4	366	One-sample t-test	0.0945	No	683.11	g=0.69
NK	>144	Male	1	NA (Non-normal)	241.8 (IQR 0.0)	425	Wilcoxon signed-rank	1.0000	No	-183.17	r_rb=NA
NK	>144	Female	5	0.8602 (Normal)	666.5 ± 167.6	404	One-sample t-test	0.0248	Yes	262.48	g=1.14

*Significant difference defined as $p < 0.05$. Values are mean ± SD if approximately normal; otherwise median (IQR). Normality assessed by Shapiro–Wilk test. One-sample t-test used for normal data; Wilcoxon signed-rank test for non-normal data. Abbreviations: T, T lymphocyte; B, B lymphocyte; NK, natural killer cell; Ig, immunoglobulin; C3/C4, complement components.

Table S6. Humoral immune indices and complement components by age and sex group.

Indicator	Age group (months)	Sex	n	Normality p (Shapiro-Wilk)	Summary statistic	Reference mean	Test	p value	Significant vs reference*	Difference vs reference	Effect size
IgG	0-12	All	67	0.0002 (Non-normal)	5.3 (IQR 4.0)	5.57	Wilcoxon signed-rank	0.5079	No	0.26	r_rb=0.09
IgG	12-24	All	41	0.0233 (Non-normal)	6.4 (IQR 5.0)	6.34	Wilcoxon signed-rank	0.6316	No	0.28	r_rb=0.09
IgG	24-36	All	24	0.7300 (Normal)	6.6 ± 2.2	7.35	One-sample t-test	0.1357	No	-0.71	g=-0.30

IgG	36-48	All	20	0.4273 (Normal)	6.3 ± 2.2	8.12	One-sample t-test	0.0012	Yes	-1.86	g=-0.81
IgG	48-60	All	10	0.4075 (Normal)	6.1 ± 2.3	8.88	One-sample t-test	0.0042	Yes	-2.82	g=-1.09
IgG	60-72	All	15	0.6370 (Normal)	5.8 ± 2.7	9.56	One-sample t-test	0.0001	Yes	-3.81	g=-1.32
IgG	72-84	All	10	0.0167 (Non-normal)	5.8 (IQR 4.5)	10.17	Wilcoxon signed-rank	0.0645	No	-3.68	r_rb=-0.67
IgG	84-96	All	4	0.2825 (Normal)	9.3 ± 8.0	10.48	One-sample t-test	0.7889	No	-1.17	g=-0.08
IgG	96-108	All	5	0.2544 (Normal)	7.6 ± 3.7	10.74	One-sample t-test	0.1299	No	-3.19	g=-0.62
IgG	108-120	All	4	0.6724 (Normal)	5.4 ± 2.0	10.86	One-sample t-test	0.0122	Yes	-5.49	g=-1.55
IgA	0-12	All	59	<0.0001 (Non-normal)	0.4 (IQR 0.4)	0.38	Wilcoxon signed-rank	0.7598	No	-0.02	r_rb=-0.06

IgA	12-24	All	40	<0.0001 (Non-normal)	0.3 (IQR 0.7)	0.47	Wilcoxon signed-rank	0.5908	No	0.06	r_rb=0.10
IgA	24-36	All	23	<0.0001 (Non-normal)	0.4 (IQR 0.3)	0.63	Wilcoxon signed-rank	0.1710	No	-0.25	r_rb=-0.45
IgA	36-48	All	19	0.0002 (Non-normal)	0.3 (IQR 0.2)	0.82	Wilcoxon signed-rank	0.0025	Yes	-0.54	r_rb=-1.00
IgA	48-60	All	9	0.3361 (Normal)	0.4 ± 0.3	1.05	One-sample t-test	<0.0001	Yes	-0.79	g=-3.80
IgA	60-72	All	12	0.1019 (Normal)	0.8 ± 0.7	1.23	One-sample t-test	0.0500	Yes	-0.44	g=-0.59
IgA	72-84	All	10	0.0182 (Non-normal)	0.4 (IQR 0.9)	1.34	Wilcoxon signed-rank	0.0273	Yes	-0.73	r_rb=-0.78
IgA	84-96	All	3	0.0472 (Non-normal)	0.3 (IQR 1.4)	1.37	Wilcoxon signed-rank	1.0000	No	-0.43	r_rb=0.00
IgA	96-108	All	5	0.1419 (Normal)	0.5 ± 0.3	1.45	One-sample t-test	0.0032	Yes	-0.98	g=-2.05

IgA	108-120	All	4	0.4683 (Normal)	0.3 ± 0.2	1.49	One-sample t-test	0.0021	Yes	-1.15	g=-2.88
IgM	0-12	All	67	0.0004 (Non-normal)	0.6 (IQR 0.5)	0.85	Wilcoxon signed-rank	0.0024	Yes	-0.18	r_rb=-0.47
IgM	12-24	All	41	0.0174 (Non-normal)	0.7 (IQR 0.4)	0.92	Wilcoxon signed-rank	0.0013	Yes	-0.22	r_rb=-0.58
IgM	24-36	All	24	0.0120 (Non-normal)	0.8 (IQR 0.4)	1.03	Wilcoxon signed-rank	0.0633	No	-0.22	r_rb=-0.43
IgM	36-48	All	20	0.0471 (Non-normal)	0.7 (IQR 0.2)	1.08	Wilcoxon signed-rank	0.0010	Yes	-0.34	r_rb=-0.84
IgM	48-60	All	10	0.1020 (Normal)	0.7 ± 0.5	1.13	One-sample t-test	0.0480	Yes	-0.39	g=-0.65
IgM	60-120	Male	28	0.0085 (Non-normal)	0.7 (IQR 0.4)	1.1	Wilcoxon signed-rank	<0.0001	Yes	-0.49	r_rb=-0.96
IgM	60-120	Female	10	0.6712 (Normal)	0.8 ± 0.5	1.33	One-sample t-test	0.0077	Yes	-0.50	g=-0.98

C3	0-12	All	30	0.0007 (Non-normal)	0.8 (IQR 0.2)	1.06	Wilcoxon signed-rank	0.0008	Yes	-0.21	r_rb=-0.70
C3	12-24	All	19	0.1595 (Normal)	1.0 ± 0.1	1.11	One-sample t-test	0.0006	Yes	-0.13	g=-0.91
C3	24-120	All	37	0.3505 (Normal)	1.0 ± 0.2	1.14	One-sample t-test	<0.0001	Yes	-0.19	g=-1.00
C4	0-12	All	30	0.0004 (Non-normal)	0.1 (IQR 0.1)	0.22	Wilcoxon signed-rank	0.0004	Yes	-0.07	r_rb=-0.75
C4	12-24	All	19	0.0573 (Normal)	0.2 ± 0.1	0.22	One-sample t-test	0.0581	No	-0.03	g=-0.44
C4	24-120	All	37	0.1951 (Normal)	0.2 ± 0.1	0.23	One-sample t-test	<0.0001	Yes	-0.05	g=-0.88

*Significant difference defined as $p < 0.05$. Values are mean ± SD if approximately normal; otherwise median (IQR). Normality assessed by Shapiro–Wilk test. One-sample t-test used for normal data; Wilcoxon signed-rank test for non-normal data. Abbreviations: T, T lymphocyte; B, B lymphocyte; NK, natural killer cell; Ig, immunoglobulin; C3/C4, complement components.

Effect sizes: For one-sample t-tests, Hedges' g (standardized mean difference) is reported; for Wilcoxon signed-rank tests, rank-biserial correlation (r_rb) is reported. "Difference vs reference" is the mean difference (t-test) or Hodges–Lehmann shift estimate (Wilcoxon).

Table S7 (1). C3 descriptive statistics by group.

Overall test: Kruskal–Wallis p=0.0986; non-normal distribution detected (≥1 group).

Indicator	Group	n	Normality p (Shapiro-Wilk)	Summary statistic
C3	Group1	15	0.1921 (Normal)	0.97 ± 0.13
C3	Group2	15	0.0858 (Normal)	0.87 ± 0.18
C3	Group3	15	0.3240 (Normal)	0.99 ± 0.23
C3	Group4	13	0.2933 (Normal)	0.87 ± 0.18
C3	Group5	9	0.5520 (Normal)	0.87 ± 0.17
C3	Group6	4	0.1629 (Normal)	1.07 ± 0.25
C3	Group7	1	NA	1.30 (IQR 0.00)
C3	Group8	1	NA	1.00 (IQR 0.00)
C3	Group9	14	0.1846 (Normal)	0.96 ± 0.16

Table S7 (2). Pairwise post-hoc comparisons for C3 (Bonferroni-adjusted) with effect sizes.

Indicator	Comparison	n1	n2	Test	Difference	Effect size	p (unadjusted)	p (Bonferroni)	Significant (Bonferroni)
C3	Group1 vs Group2	15	15	Mann–Whitney U	0.14	δ=0.44	0.0419	0.8800	No
C3	Group1 vs Group3	15	15	Mann–Whitney U	-0.04	δ=-0.15	0.4933	1.0000	No
C3	Group1 vs Group4	15	13	Mann–Whitney U	0.13	δ=0.39	0.0797	1.0000	No
C3	Group1 vs Group5	15	9	Mann–Whitney U	0.09	δ=0.33	0.1891	1.0000	No
C3	Group1 vs Group6	15	4	Mann–Whitney U	-0.01	δ=-0.03	0.9600	1.0000	No
C3	Group1 vs Group7	15	1	NA	NA	NA	NA	NA	NA
C3	Group1 vs Group8	15	1	NA	NA	NA	NA	NA	NA
C3	Group1 vs Group9	15	14	Mann–Whitney U	0.03	δ=0.13	0.5700	1.0000	No
C3	Group2 vs Group3	15	15	Mann–Whitney U	-0.16	δ=-0.42	0.0512	1.0000	No
C3	Group2 vs Group4	15	13	Mann–Whitney U	0.00	δ=-0.03	0.9265	1.0000	No
C3	Group2 vs Group5	15	9	Mann–Whitney U	-0.03	δ=-0.06	0.8345	1.0000	No

C3	Group2 vs Group6	15	4	Mann–Whitney U	-0.17	δ=-0.58	0.0887	1.0000	No
C3	Group2 vs Group7	15	1	NA	NA	NA	NA	NA	NA
C3	Group2 vs Group8	15	1	NA	NA	NA	NA	NA	NA
C3	Group2 vs Group9	15	14	Mann–Whitney U	-0.10	δ=-0.39	0.0769	1.0000	No
C3	Group3 vs Group4	15	13	Mann–Whitney U	0.15	δ=0.39	0.0799	1.0000	No
C3	Group3 vs Group5	15	9	Mann–Whitney U	0.13	δ=0.39	0.1206	1.0000	No
C3	Group3 vs Group6	15	4	Mann–Whitney U	-0.01	δ=-0.05	0.9203	1.0000	No
C3	Group3 vs Group7	15	1	NA	NA	NA	NA	NA	NA
C3	Group3 vs Group8	15	1	NA	NA	NA	NA	NA	NA
C3	Group3 vs Group9	15	14	Mann–Whitney U	0.06	δ=0.18	0.4316	1.0000	No
C3	Group4 vs Group5	13	9	Mann–Whitney U	0.00	δ=-0.01	1.0000	1.0000	No
C3	Group4 vs Group6	13	4	Mann–Whitney U	-0.17	δ=-0.62	0.0791	1.0000	No
C3	Group4 vs Group7	13	1	NA	NA	NA	NA	NA	NA
C3	Group4 vs Group8	13	1	NA	NA	NA	NA	NA	NA
C3	Group4 vs Group9	13	14	Mann–Whitney U	-0.10	δ=-0.34	0.1385	1.0000	No

C3	Group5 vs Group6	9	4	Mann–Whitney U	-0.18	δ=-0.44	0.2601	1.0000	No
C3	Group5 vs Group7	9	1	NA	NA	NA	NA	NA	NA
C3	Group5 vs Group8	9	1	NA	NA	NA	NA	NA	NA
C3	Group5 vs Group9	9	14	Mann–Whitney U	-0.09	δ=-0.30	0.2436	1.0000	No
C3	Group6 vs Group7	4	1	NA	NA	NA	NA	NA	NA
C3	Group6 vs Group8	4	1	NA	NA	NA	NA	NA	NA
C3	Group6 vs Group9	4	14	Mann–Whitney U	0.08	δ=0.29	0.4248	1.0000	No
C3	Group7 vs Group8	1	1	NA	NA	NA	NA	NA	NA
C3	Group7 vs Group9	1	14	NA	NA	NA	NA	NA	NA
C3	Group8 vs Group9	1	14	NA	NA	NA	NA	NA	NA

Table S8 (1). C4 descriptive statistics by group.

Overall test: Kruskal–Wallis p=0.7151; non-normal distribution detected (≥1 group).

Indicator	Group	n	Normality p (Shapiro-Wilk)	Summary statistic
C4	Group1	15	0.2226 (Normal)	0.17 ± 0.05
C4	Group2	15	0.9873 (Normal)	0.16 ± 0.03
C4	Group3	15	0.7255 (Normal)	0.18 ± 0.06
C4	Group4	13	0.4371 (Normal)	0.16 ± 0.05
C4	Group5	9	0.0950 (Normal)	0.17 ± 0.07
C4	Group6	4	0.4301 (Normal)	0.23 ± 0.12
C4	Group7	1	NA	0.27 (IQR 0.00)
C4	Group8	1	NA	0.22 (IQR 0.00)
C4	Group9	14	0.5163 (Normal)	0.20 ± 0.07

Table S8 (2). Pairwise post-hoc comparisons for C4 (Bonferroni-adjusted) with effect sizes.

Indicator	Comparison	n1	n2	Test	Difference	Effect size	p (unadjusted)	p (Bonferroni)	Significant (Bonferroni)
C4	Group1 vs Group2	15	15	Mann–Whitney U	0.00	δ=0.04	0.8679	1.0000	No
C4	Group1 vs Group3	15	15	Mann–Whitney U	-0.01	δ=-0.08	0.7234	1.0000	No
C4	Group1 vs Group4	15	13	Mann–Whitney U	0.00	δ=0.01	0.9815	1.0000	No

C4	Group1 vs Group5	15	9	Mann–Whitney U	0.00	δ=0.00	1.0000	1.0000	No
C4	Group1 vs Group6	15	4	Mann–Whitney U	-0.04	δ=-0.32	0.3664	1.0000	No
C4	Group1 vs Group7	15	1	NA	NA	NA	NA	NA	NA
C4	Group1 vs Group8	15	1	NA	NA	NA	NA	NA	NA
C4	Group1 vs Group9	15	14	Mann–Whitney U	-0.03	δ=-0.28	0.2127	1.0000	No
C4	Group2 vs Group3	15	15	Mann–Whitney U	-0.02	δ=-0.20	0.3710	1.0000	No
C4	Group2 vs Group4	15	13	Mann–Whitney U	0.00	δ=0.00	1.0000	1.0000	No
C4	Group2 vs Group5	15	9	Mann–Whitney U	0.00	δ=0.01	1.0000	1.0000	No
C4	Group2 vs Group6	15	4	Mann–Whitney U	-0.07	δ=-0.23	0.5138	1.0000	No
C4	Group2 vs Group7	15	1	NA	NA	NA	NA	NA	NA
C4	Group2 vs Group8	15	1	NA	NA	NA	NA	NA	NA
C4	Group2 vs Group9	15	14	Mann–Whitney U	-0.03	δ=-0.34	0.1198	1.0000	No
C4	Group3 vs Group4	15	13	Mann–Whitney U	0.01	δ=0.15	0.5022	1.0000	No
C4	Group3 vs Group5	15	9	Mann–Whitney U	0.01	δ=0.09	0.7422	1.0000	No

C4	Group3 vs Group6	15	4	Mann–Whitney U	-0.04	δ=-0.22	0.5466	1.0000	No
C4	Group3 vs Group7	15	1	NA	NA	NA	NA	NA	NA
C4	Group3 vs Group8	15	1	NA	NA	NA	NA	NA	NA
C4	Group3 vs Group9	15	14	Mann–Whitney U	-0.02	δ=-0.16	0.4707	1.0000	No
C4	Group4 vs Group5	13	9	Mann–Whitney U	0.00	δ=0.02	0.9732	1.0000	No
C4	Group4 vs Group6	13	4	Mann–Whitney U	-0.04	δ=-0.29	0.4249	1.0000	No
C4	Group4 vs Group7	13	1	NA	NA	NA	NA	NA	NA
C4	Group4 vs Group8	13	1	NA	NA	NA	NA	NA	NA
C4	Group4 vs Group9	13	14	Mann–Whitney U	-0.03	δ=-0.32	0.1581	1.0000	No
C4	Group5 vs Group6	9	4	Mann–Whitney U	-0.03	δ=-0.28	0.4856	1.0000	No
C4	Group5 vs Group7	9	1	NA	NA	NA	NA	NA	NA
C4	Group5 vs Group8	9	1	NA	NA	NA	NA	NA	NA
C4	Group5 vs Group9	9	14	Mann–Whitney U	-0.03	δ=-0.25	0.3441	1.0000	No
C4	Group6 vs Group7	4	1	NA	NA	NA	NA	NA	NA
C4	Group6 vs Group8	4	1	NA	NA	NA	NA	NA	NA
C4	Group6 vs Group9	4	14	Mann–Whitney U	0.01	δ=0.09	0.8316	1.0000	No
C4	Group7 vs Group8	1	1	NA	NA	NA	NA	NA	NA

C4	Group7 vs Group9	1	14	NA	NA	NA	NA	NA	NA
C4	Group8 vs Group9	1	14	NA	NA	NA	NA	NA	NA

Table S9 (1). IgA descriptive statistics by group.

Overall test: Kruskal–Wallis p=0.0005; non-normal distribution detected (≥1 group).

Indicator	Group	n	Normality p (Shapiro-Wilk)	Summary statistic
IgA	Group1	36	<0.0001 (Non-normal)	0.31 (IQR 0.36)
IgA	Group2	36	<0.0001 (Non-normal)	0.31 (IQR 0.29)
IgA	Group3	35	0.0044 (Non-normal)	0.58 (IQR 0.90)
IgA	Group4	17	0.0134 (Non-normal)	0.29 (IQR 0.43)
IgA	Group5	21	0.0002 (Non-normal)	0.21 (IQR 0.22)
IgA	Group6	13	0.1013 (Normal)	1.04 ± 0.84
IgA	Group7	7	0.0095 (Non-normal)	0.49 (IQR 0.70)
IgA	Group8	2	NA	0.47 (IQR 0.27)
IgA	Group9	22	0.0003 (Non-normal)	0.37 (IQR 0.49)

Table S9 (2). Pairwise post-hoc comparisons for IgA (Bonferroni-adjusted) with effect sizes.

Indicator	Comparison	n1	n2	Test	Difference	Effect size	p (unadjusted)	p (Bonferroni)	Significant (Bonferroni)
IgA	Group1 vs Group2	36	36	Mann–Whitney U	0.03	δ=0.08	0.5580	1.0000	No
IgA	Group1 vs Group3	36	35	Mann–Whitney U	-0.26	δ=-0.38	0.0065	0.2348	No
IgA	Group1 vs Group4	36	17	Mann–Whitney U	0.01	δ=0.02	0.9089	1.0000	No
IgA	Group1 vs Group5	36	21	Mann–Whitney U	0.09	δ=0.26	0.1014	1.0000	No
IgA	Group1 vs Group6	36	13	Mann–Whitney U	-0.45	δ=-0.45	0.0168	0.6062	No
IgA	Group1 vs Group7	36	7	Mann–Whitney U	-0.26	δ=-0.53	0.0286	1.0000	No
IgA	Group1 vs Group8	36	2	Mann–Whitney U	-0.04	δ=-0.06	0.9219	1.0000	No
IgA	Group1 vs Group9	36	22	Mann–Whitney U	-0.01	δ=-0.03	0.8663	1.0000	No
IgA	Group2 vs Group3	36	35	Mann–Whitney U	-0.30	δ=-0.46	0.0010	0.0346	Yes
IgA	Group2 vs Group4	36	17	Mann–Whitney U	-0.02	δ=-0.05	0.7822	1.0000	No

IgA	Group2 vs Group5	36	21	Mann–Whitney U	0.05	δ=0.21	0.1996	1.0000	No
IgA	Group2 vs Group6	36	13	Mann–Whitney U	-0.54	δ=-0.52	0.0059	0.2132	No
IgA	Group2 vs Group7	36	7	Mann–Whitney U	-0.32	δ=-0.61	0.0118	0.4256	No
IgA	Group2 vs Group8	36	2	Mann–Whitney U	-0.09	δ=-0.22	0.6238	1.0000	No
IgA	Group2 vs Group9	36	22	Mann–Whitney U	-0.04	δ=-0.14	0.3693	1.0000	No
IgA	Group3 vs Group4	35	17	Mann–Whitney U	0.27	δ=0.38	0.0282	1.0000	No
IgA	Group3 vs Group5	35	21	Mann–Whitney U	0.37	δ=0.56	0.0005	0.0165	Yes
IgA	Group3 vs Group6	35	13	Mann–Whitney U	-0.11	δ=-0.12	0.5235	1.0000	No
IgA	Group3 vs Group7	35	7	Mann–Whitney U	-0.07	δ=-0.07	0.8001	1.0000	No
IgA	Group3 vs Group8	35	2	Mann–Whitney U	0.24	δ=0.27	0.5455	1.0000	No
IgA	Group3 vs Group9	35	22	Mann–Whitney U	0.23	δ=0.31	0.0501	1.0000	No
IgA	Group4 vs Group5	17	21	Mann–Whitney U	0.09	δ=0.22	0.2643	1.0000	No

IgA	Group4 vs Group6	17	13	Mann–Whitney U	-0.43	δ=-0.48	0.0280	1.0000	No
IgA	Group4 vs Group7	17	7	Mann–Whitney U	-0.29	δ=-0.51	0.0566	1.0000	No
IgA	Group4 vs Group8	17	2	Mann–Whitney U	-0.07	δ=-0.12	0.8420	1.0000	No
IgA	Group4 vs Group9	17	22	Mann–Whitney U	-0.02	δ=-0.05	0.7877	1.0000	No
IgA	Group5 vs Group6	21	13	Mann–Whitney U	-0.56	δ=-0.66	0.0014	0.0512	No
IgA	Group5 vs Group7	21	7	Mann–Whitney U	-0.37	δ=-0.70	0.0068	0.2439	No
IgA	Group5 vs Group8	21	2	Mann–Whitney U	-0.10	δ=-0.33	0.4780	1.0000	No
IgA	Group5 vs Group9	21	22	Mann–Whitney U	-0.09	δ=-0.28	0.1141	1.0000	No
IgA	Group6 vs Group7	13	7	Mann–Whitney U	-0.01	δ=-0.01	1.0000	1.0000	No
IgA	Group6 vs Group8	13	2	Mann–Whitney U	0.40	δ=0.46	0.3810	1.0000	No
IgA	Group6 vs Group9	13	22	Mann–Whitney U	0.36	δ=0.40	0.0558	1.0000	No
IgA	Group7 vs Group8	7	2	Mann–Whitney U	0.28	δ=0.29	0.6592	1.0000	No

IgA	Group7 vs Group9	7	22	Mann–Whitney U	0.31	δ=0.44	0.0876	1.0000	No
IgA	Group8 vs Group9	2	22	Mann–Whitney U	0.04	δ=0.18	0.7145	1.0000	No

Table S10 (1). IgG descriptive statistics by group.

Overall test: Kruskal–Wallis p=<0.0001; non-normal distribution detected (≥1 group).

Indicator	Group	n	Normality p (Shapiro-Wilk)	Summary statistic
IgG	Group1	39	0.0814 (Normal)	6.18 ± 2.24
IgG	Group2	39	<0.0001 (Non-normal)	4.81 (IQR 2.94)
IgG	Group3	36	0.4067 (Normal)	7.99 ± 3.13
IgG	Group4	22	0.4706 (Normal)	5.38 ± 2.36
IgG	Group5	22	0.1430 (Normal)	5.27 ± 2.14
IgG	Group6	13	0.9872 (Normal)	8.40 ± 2.68
IgG	Group7	7	0.6278 (Normal)	7.58 ± 2.10
IgG	Group8	2	NA	8.39 (IQR 2.11)
IgG	Group9	22	0.9527 (Normal)	7.09 ± 2.74

Table S10 (2). Pairwise post-hoc comparisons for IgG (Bonferroni-adjusted) with effect sizes.

Indicator	Comparison	n1	n2	Test	Difference	Effect size	p (unadjusted)	p (Bonferroni)	Significant (Bonferroni)
IgG	Group1 vs Group2	39	39	Mann–Whitney U	0.91	δ=0.24	0.0659	1.0000	No
IgG	Group1 vs Group3	39	36	Mann–Whitney U	-1.89	δ=-0.34	0.0106	0.3814	No
IgG	Group1 vs Group4	39	22	Mann–Whitney U	0.83	δ=0.21	0.1863	1.0000	No
IgG	Group1 vs Group5	39	22	Mann–Whitney U	0.91	δ=0.22	0.1493	1.0000	No
IgG	Group1 vs Group6	39	13	Mann–Whitney U	-2.23	δ=-0.48	0.0106	0.3801	No
IgG	Group1 vs Group7	39	7	Mann–Whitney U	-1.44	δ=-0.38	0.1132	1.0000	No
IgG	Group1 vs Group8	39	2	Mann–Whitney U	-2.34	δ=-0.49	0.2951	1.0000	No
IgG	Group1 vs Group9	39	22	Mann–Whitney U	-1.03	δ=-0.22	0.1515	1.0000	No
IgG	Group2 vs Group3	39	36	Mann–Whitney U	-2.82	δ=-0.49	0.0003	0.0105	Yes
IgG	Group2 vs Group4	39	22	Mann–Whitney U	-0.14	δ=-0.03	0.8628	1.0000	No

IgG	Group2 vs Group5	39	22	Mann–Whitney U	-0.05	δ=-0.01	0.9342	1.0000	No
IgG	Group2 vs Group6	39	13	Mann–Whitney U	-3.24	δ=-0.61	0.0011	0.0394	Yes
IgG	Group2 vs Group7	39	7	Mann–Whitney U	-2.39	δ=-0.58	0.0171	0.6141	No
IgG	Group2 vs Group8	39	2	Mann–Whitney U	-3.20	δ=-0.62	0.1549	1.0000	No
IgG	Group2 vs Group9	39	22	Mann–Whitney U	-2.00	δ=-0.39	0.0124	0.4461	No
IgG	Group3 vs Group4	36	22	Mann–Whitney U	2.68	δ=0.48	0.0021	0.0773	No
IgG	Group3 vs Group5	36	22	Mann–Whitney U	2.90	δ=0.51	0.0012	0.0435	Yes
IgG	Group3 vs Group6	36	13	Mann–Whitney U	-0.37	δ=-0.08	0.6835	1.0000	No
IgG	Group3 vs Group7	36	7	Mann–Whitney U	0.55	δ=0.09	0.7298	1.0000	No
IgG	Group3 vs Group8	36	2	Mann–Whitney U	-0.74	δ=-0.06	0.9219	1.0000	No
IgG	Group3 vs Group9	36	22	Mann–Whitney U	0.79	δ=0.15	0.3323	1.0000	No
IgG	Group4 vs Group5	22	22	Mann–Whitney U	0.08	δ=0.02	0.8973	1.0000	No

IgG	Group4 vs Group6	22	13	Mann–Whitney U	-3.12	δ=-0.60	0.0033	0.1195	No
IgG	Group4 vs Group7	22	7	Mann–Whitney U	-2.17	δ=-0.51	0.0497	1.0000	No
IgG	Group4 vs Group8	22	2	Mann–Whitney U	-2.90	δ=-0.59	0.1916	1.0000	No
IgG	Group4 vs Group9	22	22	Mann–Whitney U	-1.82	δ=-0.37	0.0378	1.0000	No
IgG	Group5 vs Group6	22	13	Mann–Whitney U	-3.13	δ=-0.67	0.0012	0.0425	Yes
IgG	Group5 vs Group7	22	7	Mann–Whitney U	-2.36	δ=-0.60	0.0178	0.6400	No
IgG	Group5 vs Group8	22	2	Mann–Whitney U	-3.36	δ=-0.77	0.0870	1.0000	No
IgG	Group5 vs Group9	22	22	Mann–Whitney U	-1.98	δ=-0.42	0.0189	0.6807	No
IgG	Group6 vs Group7	13	7	Mann–Whitney U	0.94	δ=0.16	0.5880	1.0000	No
IgG	Group6 vs Group8	13	2	Mann–Whitney U	0.16	δ=0.00	1.0000	1.0000	No
IgG	Group6 vs Group9	13	22	Mann–Whitney U	1.24	δ=0.24	0.2527	1.0000	No
IgG	Group7 vs Group8	7	2	Mann–Whitney U	-0.87	δ=-0.14	0.8889	1.0000	No

IgG	Group7 vs Group9	7	22	Mann–Whitney U	0.27	δ=0.06	0.8227	1.0000	No
IgG	Group8 vs Group9	2	22	Mann–Whitney U	1.46	δ=0.23	0.6522	1.0000	No

Table S11 (1). IgM descriptive statistics by group.

Overall test: Kruskal–Wallis p=0.0064; non-normal distribution detected (≥1 group).

Indicator	Group	n	Normality p (Shapiro-Wilk)	Summary statistic
IgM	Group1	30	0.0073 (Non-normal)	0.68 (IQR 0.42)
IgM	Group2	30	0.0042 (Non-normal)	0.61 (IQR 0.29)
IgM	Group3	24	0.2473 (Normal)	0.96 ± 0.36
IgM	Group4	16	0.8235 (Normal)	0.65 ± 0.28
IgM	Group5	20	0.0016 (Non-normal)	0.51 (IQR 0.38)
IgM	Group6	13	0.0148 (Non-normal)	0.74 (IQR 0.30)
IgM	Group7	5	0.5231 (Normal)	1.27 ± 0.63
IgM	Group8	1	NA	1.11 (IQR 0.00)
IgM	Group9	16	0.1189 (Normal)	0.75 ± 0.42

Table S11 (2). Pairwise post-hoc comparisons for IgM (Bonferroni-adjusted) with effect sizes.

Indicator	Comparison	n1	n2	Test	Difference	Effect size	p (unadjusted)	p (Bonferroni)	Significant (Bonferroni)
IgM	Group1 vs Group2	30	30	Mann–Whitney U	0.01	δ=0.03	0.8650	1.0000	No
IgM	Group1 vs Group3	30	24	Mann–Whitney U	-0.25	δ=-0.39	0.0148	0.4142	No
IgM	Group1 vs Group4	30	16	Mann–Whitney U	0.05	δ=0.10	0.5720	1.0000	No
IgM	Group1 vs Group5	30	20	Mann–Whitney U	0.08	δ=0.20	0.2466	1.0000	No
IgM	Group1 vs Group6	30	13	Mann–Whitney U	-0.14	δ=-0.30	0.1218	1.0000	No
IgM	Group1 vs Group7	30	5	Mann–Whitney U	-0.46	δ=-0.57	0.0451	1.0000	No
IgM	Group1 vs Group8	30	1	NA	NA	NA	NA	NA	NA
IgM	Group1 vs Group9	30	16	Mann–Whitney U	0.00	δ=0.00	1.0000	1.0000	No
IgM	Group2 vs Group3	30	24	Mann–Whitney U	-0.25	δ=-0.44	0.0064	0.1803	No
IgM	Group2 vs Group4	30	16	Mann–Whitney U	0.03	δ=0.06	0.7380	1.0000	No
IgM	Group2 vs Group5	30	20	Mann–Whitney U	0.10	δ=0.19	0.2674	1.0000	No

IgM	Group2 vs Group6	30	13	Mann–Whitney U	-0.15	δ=-0.34	0.0833	1.0000	No
IgM	Group2 vs Group7	30	5	Mann–Whitney U	-0.49	δ=-0.61	0.0287	0.8025	No
IgM	Group2 vs Group8	30	1	NA	NA	NA	NA	NA	NA
IgM	Group2 vs Group9	30	16	Mann–Whitney U	-0.01	δ=-0.02	0.9357	1.0000	No
IgM	Group3 vs Group4	24	16	Mann–Whitney U	0.31	δ=0.48	0.0120	0.3353	No
IgM	Group3 vs Group5	24	20	Mann–Whitney U	0.31	δ=0.53	0.0027	0.0742	No
IgM	Group3 vs Group6	24	13	Mann–Whitney U	0.09	δ=0.15	0.4642	1.0000	No
IgM	Group3 vs Group7	24	5	Mann–Whitney U	-0.17	δ=-0.28	0.3404	1.0000	No
IgM	Group3 vs Group8	24	1	NA	NA	NA	NA	NA	NA
IgM	Group3 vs Group9	24	16	Mann–Whitney U	0.25	δ=0.35	0.0643	1.0000	No
IgM	Group4 vs Group5	16	20	Mann–Whitney U	0.03	δ=0.07	0.7502	1.0000	No
IgM	Group4 vs Group6	16	13	Mann–Whitney U	-0.19	δ=-0.37	0.1001	1.0000	No
IgM	Group4 vs Group7	16	5	Mann–Whitney U	-0.52	δ=-0.65	0.0318	0.8916	No
IgM	Group4 vs Group8	16	1	NA	NA	NA	NA	NA	NA

IgM	Group4 vs Group9	16	16	Mann–Whitney U	-0.05	δ=-0.08	0.7203	1.0000	No
IgM	Group5 vs Group6	20	13	Mann–Whitney U	-0.24	δ=-0.51	0.0158	0.4427	No
IgM	Group5 vs Group7	20	5	Mann–Whitney U	-0.50	δ=-0.74	0.0096	0.2677	No
IgM	Group5 vs Group8	20	1	NA	NA	NA	NA	NA	NA
IgM	Group5 vs Group9	20	16	Mann–Whitney U	-0.07	δ=-0.14	0.4737	1.0000	No
IgM	Group6 vs Group7	13	5	Mann–Whitney U	-0.32	δ=-0.45	0.1734	1.0000	No
IgM	Group6 vs Group8	13	1	NA	NA	NA	NA	NA	NA
IgM	Group6 vs Group9	13	16	Mann–Whitney U	0.15	δ=0.23	0.3028	1.0000	No
IgM	Group7 vs Group8	5	1	NA	NA	NA	NA	NA	NA
IgM	Group7 vs Group9	5	16	Mann–Whitney U	0.46	δ=0.64	0.0389	1.0000	No
IgM	Group8 vs Group9	1	16	NA	NA	NA	NA	NA	NA

Notes: Normality assessed using Shapiro–Wilk test within each group. If all groups were approximately normal, pairwise Welch t-tests were applied; otherwise Mann–Whitney U tests were used. Bonferroni correction was applied within each indicator across all pairwise comparisons. Differences are mean differences (parametric) or Hodges–Lehmann location shifts (non-parametric). Effect sizes are Hedges' g (parametric) or Cliff's delta δ (non-parametric).

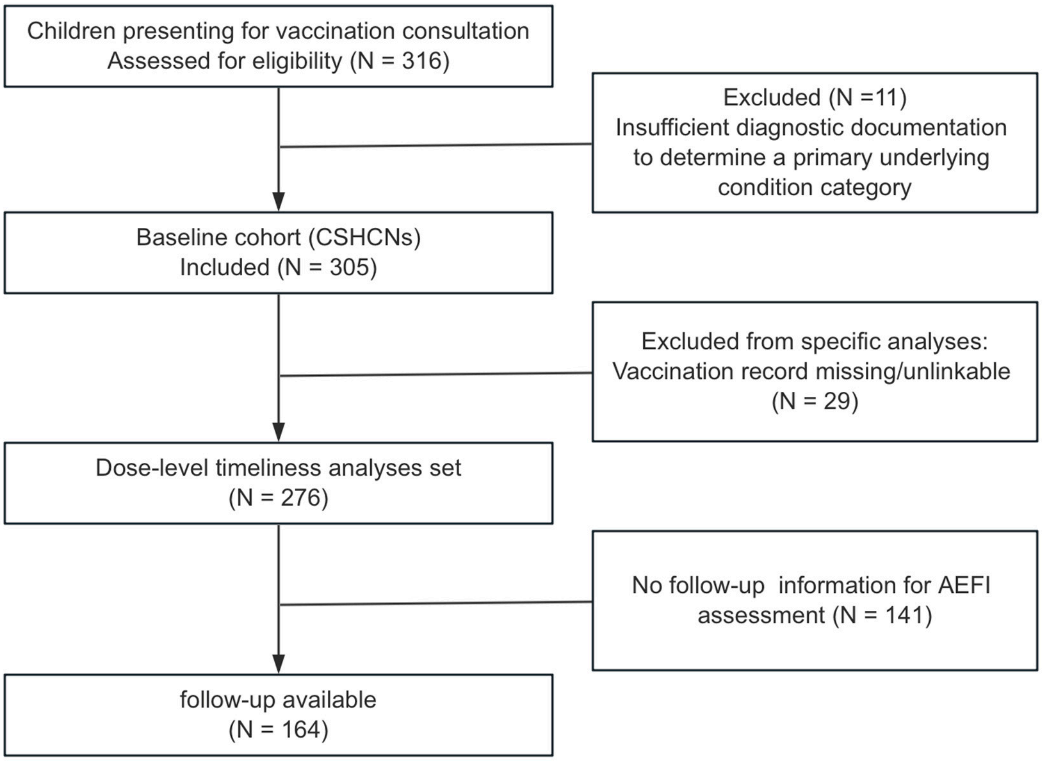

Figure S1. Study flow diagram. Children presenting for vaccination consultation between August 2019 and April 2025 were assessed for eligibility (N = 316). Eleven children were excluded because the medical chart lacked sufficient diagnostic documentation to determine a primary underlying condition category, leaving 305 children in the baseline CSHCN cohort. 29 children lacked linkable vaccination records and were excluded from timeliness calculations requiring vaccination dates; dose-level timeliness analyses were conducted among children with linkable vaccination records (N = 276); AEFI analyses were restricted to children with follow-up information available (N = 164); 141 children without follow-up information were excluded from AEFI analyses.

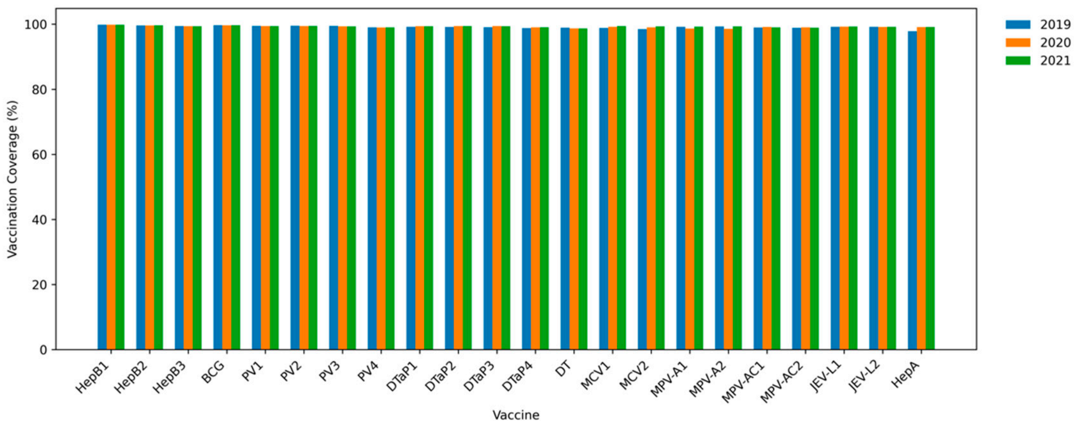

Figure S2. Vaccination coverage of National Immunization Program (NIP) vaccines by vaccine type in 2019–2021. Bars represent annual vaccination coverage for each vaccine.

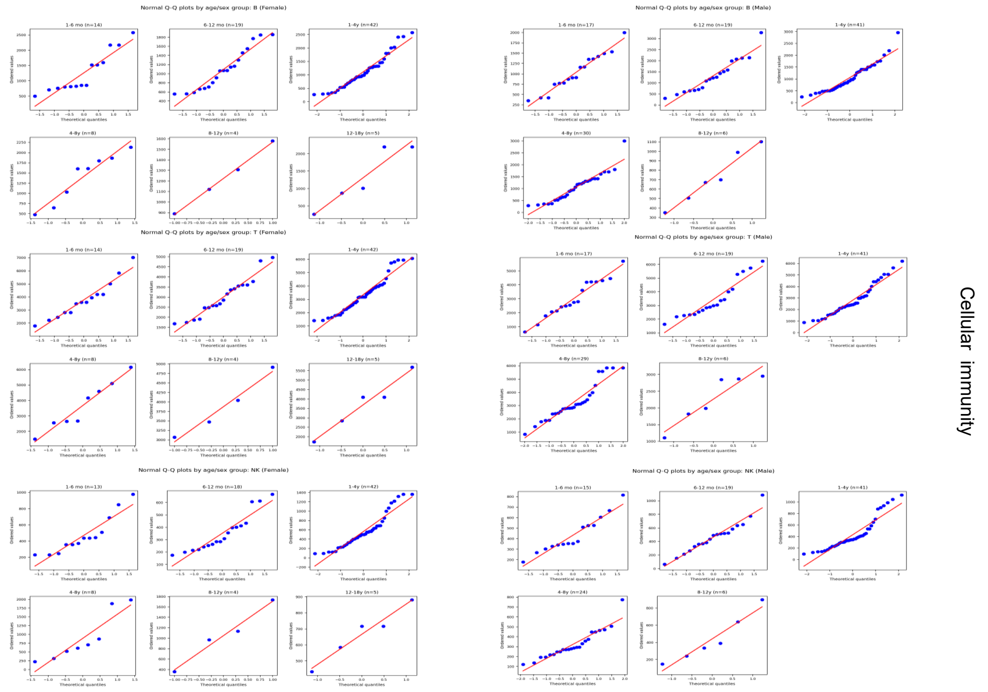
Normal Q-Q plots by age/sex group: B (Female)
Normal Q-Q plots by age/sex group: B (Male)
Normal Q-Q plots by age/sex group: T (Female)
Normal Q-Q plots by age/sex group: T (Male)
Normal Q-Q plots by age/sex group: NK (Female)
Normal Q-Q plots by age/sex group: NK (Male)
Cellular immunity

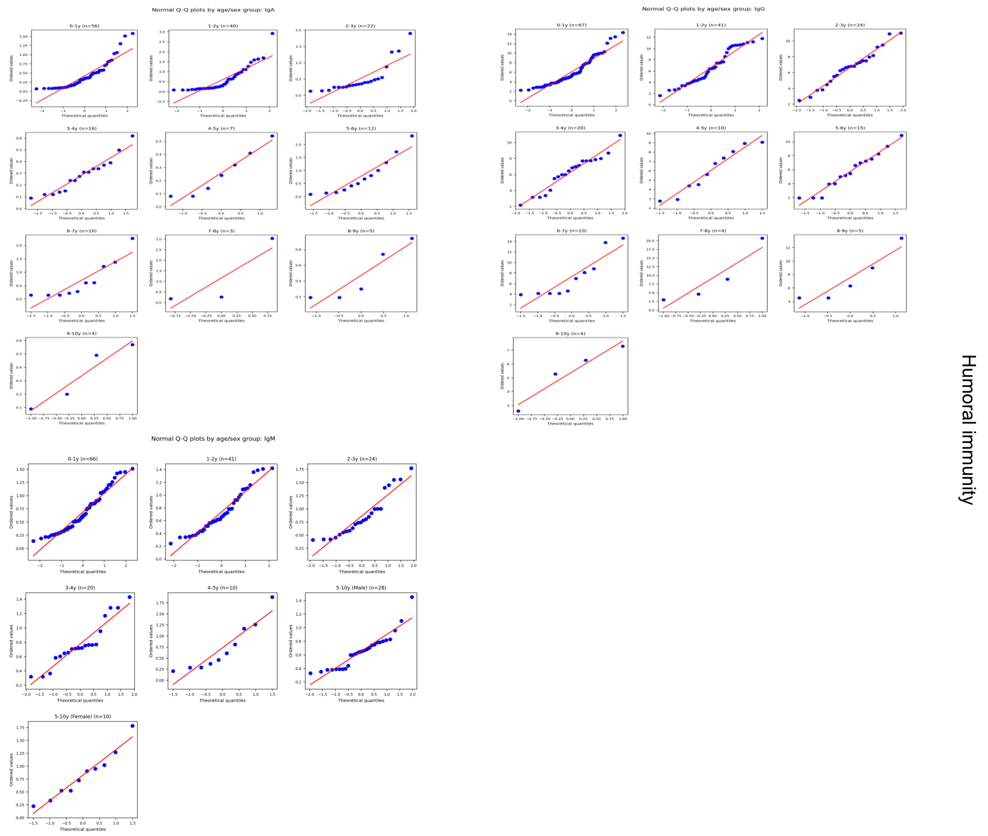
Normal Q-Q plots by age/sex group: IgA
Normal Q-Q plots by age/sex group: IgG
Normal Q-Q plots by age/sex group: IgM
Humoral immunity

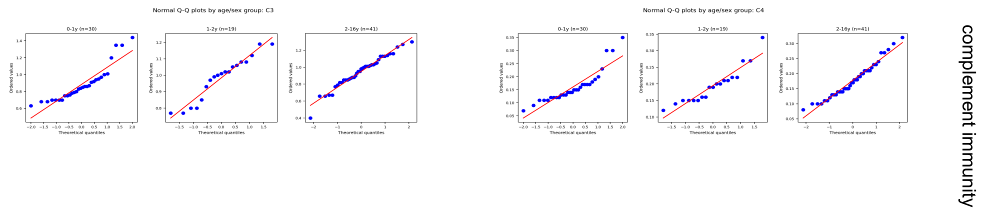
Normal Q-Q plots by age/sex group: C3
Normal Q-Q plots by age/sex group: C4
complement immunity

Figure S3. Normal Q–Q plots for immunological indicators across subgroups. Normal quantile–quantile (Q–Q) plots are shown to assess the normality of each immunological indicator within each analytic subgroup used in the comparisons (as labeled in each small panel). Blue dots represent the ordered observed values plotted against the theoretical quantiles of a normal distribution, and the red line denotes the expected relationship under normality. Systematic departures of points from the reference line indicate deviations from a normal distribution. Panels are grouped by immune domains: Cellular immunity (T-cell, B-cell, and NK-cell measures), Humoral immunity (IgG, IgA, and IgM), and Complement immunity (C3 and C4). These diagnostics were used to guide the choice of parametric versus non-parametric statistical tests and to support the appropriateness of the analytical assumptions.

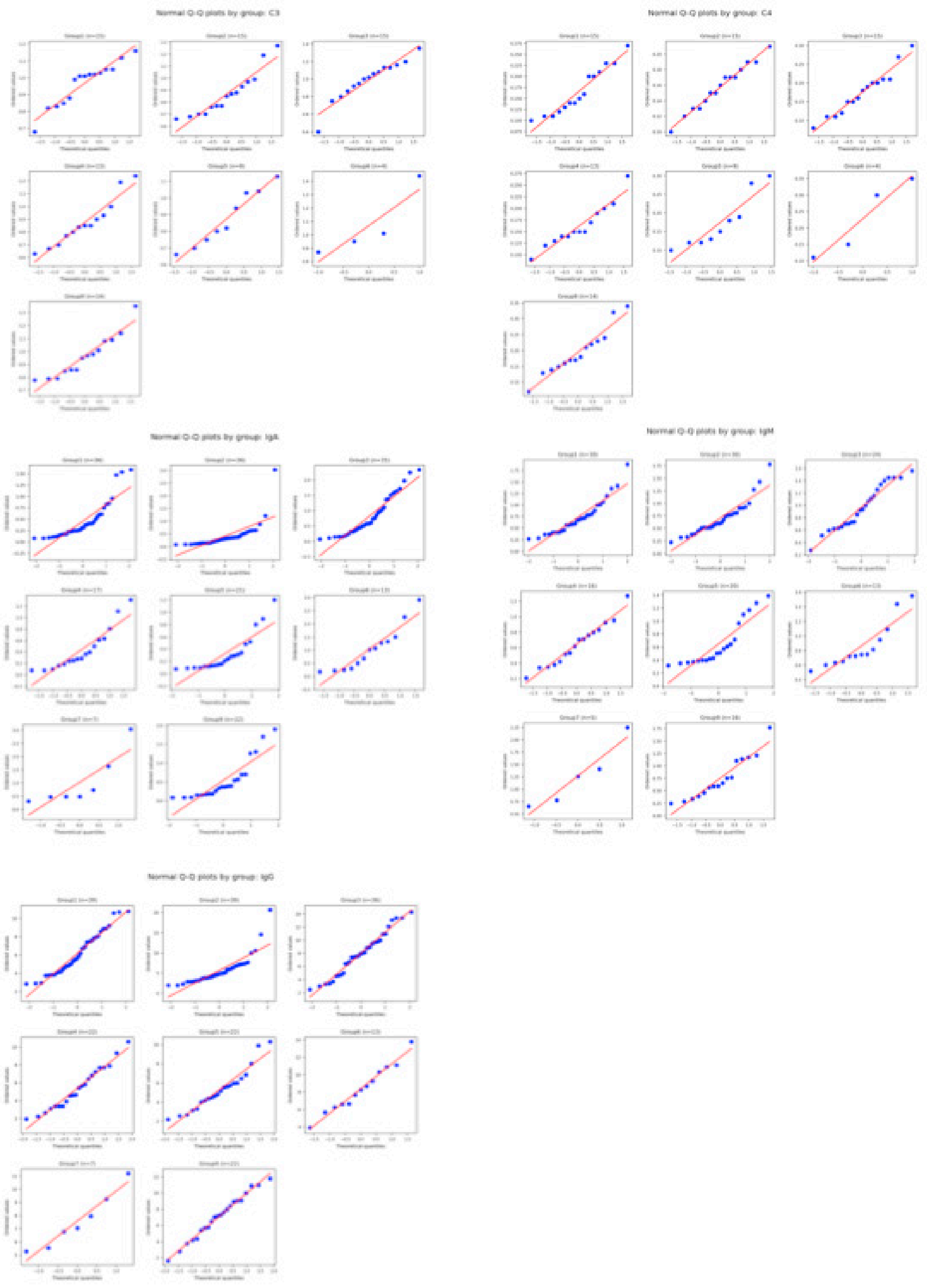

Figure S4. Normal Q–Q plots of humoral and complement immune indicators across study groups.

Normal quantile–quantile (Q–Q) plots are presented to evaluate the normality assumption for each immunological indicator within each study group. In each panel, blue points represent the ordered observed values and the red reference line indicates

the expected relationship under a theoretical normal distribution. Deviations from the reference line, particularly in the tails, suggest departures from normality (e.g., skewness or heavy tails).

Panels are organized by indicator (IgG, IgA, IgM, C3, and C4), with individual subpanels corresponding to the group comparisons analyzed in this study (group labels shown above each subpanel). These graphical diagnostics were used to inform the choice of parametric versus non-parametric tests in subsequent between-group analyses and to support assessment of model assumptions.
